# QTAIM Based Computational Assessment of Cleavage Prone Bonds in Highly Hazardous Pesticides

**DOI:** 10.3390/toxics13100839

**Published:** 2025-10-01

**Authors:** Andrés Aracena, Sebastián Elgueta, Sebastián Pizarro, César Zúñiga

**Affiliations:** 1 Instituto de Ciencias Naturales, Facultad de Medicina Veterinaria y Agronomía, Universidad de Las Américas, Sede Santiago, Campus La Florida, Avenida Walker Martínez 1360, La Florida, Santiago 8240000, Chile; 2 Centro de Investigación en Ciencias Biológicas y Químicas, Universidad de Las Américas, Avenida Manuel Montt 948, Providencia, Santiago 7500000, Chile; czuniga@udla.cl; 3 Escuela de Nutrición y Dietética, Facultad de Ciencias de la Rehabilitación y Calidad de Vida, Universidad San Sebastián, Providencia 7500000, Chile; sebastian.elgueta@uss.cl; 4 Escuela de Ingeniería Química, Pontificia Universidad Católica de Valparaíso, Avenida Brasil 2162, Valparaíso 2340000, Chile; sebastian.pizarro.a@mail.pucv.cl; 5 Instituto de Ciencias Naturales, Facultad de Medicina Veterinaria y Agronomía, Universidad de Las Américas, Sede Santiago, Campus Providencia, Avenida Manuel Montt 948, Providencia, Santiago 7500000, Chile

**Keywords:** highly hazardous pesticides, cleavage, QTAIM, TAFF, reactivity descriptors

## Abstract

Highly Hazardous Pesticides (HHPs) pose severe risks to human health and the environment, making it essential to understand their molecular stability and degradation pathways. In this study, the Quantum Theory of Atoms in Molecules (QTAIM) was applied to four representative organophosphate pesticides, allowing the identification of electronically weak bonds as intrinsic sites of lability. These findings are consistent with reported hydrolytic, oxidative, enzymatic, and microbial degradation routes. Importantly, QTAIM descriptors proved largely insensitive to solvation, confirming their intrinsic character within the molecular electronic structure. To complement QTAIM, conceptual DFT (Density Functional Theory) reactivity indices were analyzed, revealing that solvent effects induce more noticeable variations in global and local descriptors than in topological parameters. In addition, a Topological Analysis of the Fukui Function (TAFF) was performed, which mapped nucleophilic, electrophilic, and radical susceptibilities directly onto QTAIM basins. The TAFF analysis confirmed that bonds identified as weak by QTAIM (notably P–O, P–S, and P–N linkages) also coincide with the most reactive sites, thereby reinforcing their mechanistic role in degradation pathways. This integrated framework highlights the robustness of QTAIM, the sensitivity of global and local reactivity descriptors to solvation revealed by conceptual DFT, and the complementary insights provided by TAFF, contributing to risk assessment, remediation strategies, and the rational design of safer pesticides.

## 1. Introduction

Pesticides are crucial for managing pests and diseases in agricultural systems. However, their overuse and improper application in farms can result in significant hazards [1]. The widespread use of organophosphate insecticides, such as methamidophos, acephate, chlorpyriphos, and diazinon, to control pests raises substantial concerns regarding human health and ecological integrity [2]. Different evidence indicates that these substances pose acute and chronic risks; for example, methamidophos is classified by the World Health Organization as highly toxic (Class I), while acephate, chlorpyrifos, and diazinon are categorized as moderately hazardous (Class II) [1].

Numerous scientific studies have demonstrated that chlorpyrifos presents a significant concern with multifaceted toxicological implications spanning neurodevelopment [3,4,5,6]. The insecticide diazinon can cause neurodevelopmental and long-term cognitive effects, acute neurotoxicity, environmental persistence, and ecotoxicity, posing risks to aquatic organisms and other wildlife ecosystems [7,8]. Notably, methamidophos is linked to neurotoxic effects, including disruption of cholinergic neurotransmission and inhibition of acetylcholinesterase (AChE) activity [9,10,11]. Additionally, acephate poses risks to neurochemistry, development, reproduction, and the environment because of its toxicity [12,13]. Furthermore, these compounds are highly toxic to non-target organisms, especially aquatic wildlife, emphasizing the importance of developing effective strategies to reduce their lingering toxicity and environmental impacts [14]. Although these four compounds differ in their acute toxicity classes, they all share the same mechanism of acetylcholinesterase inhibition, neurotoxic potential, and environmental persistence. This makes them examples of Highly Hazardous Pesticides, which the World Health Organization (WHO) and Food and Agriculture Organization of the United Nations (FAO) classify as toxic and recommend their restriction globally [15,16].

The Quantum Theory of Atoms in Molecules (QTAIM), initially formulated by R. F. W. Bader [17,18,19], is a theoretical framework based on the topology of the electron density (ρ(r)) that enables a rigorous description of chemical bonding and molecular structure. QTAIM represents a robust and predictive framework for exploring molecular topology and reactivity, where topological descriptors correlate with bond dissociation energies, offering a fundamental foundation for interpreting bond stability and chemical reactivity [20].

Theoretical studies of structure properties-reactivity have been made in diazinon [21], chlorpyrifos [22,23,24,25,26], acephate [27,28,29,30], and methamidophos [29,31,32]. Since the studied organophosphate pesticides are frequently exposed to aqueous environments, either in agricultural applications or during environmental transport, it is crucial to evaluate the role of solvation in their electronic properties. To this end, both a dielectric continuum model (PCM with water as solvent) and a supermolecular approach including explicit water molecules within the PCM framework were employed to assess their influence on the QTAIM descriptors of bond topology.

The QTAIM provides a robust theoretical framework for molecular characterization and serves as an effective instrument for analyzing pesticide degradation mechanisms [33,34]. Given their hazard profiles, comprehending the molecular-level breakdown of these pesticides is of paramount importance [35]. QTAIM offers a comprehensive perspective on electron density, rendering it suitable for identifying bond critical points and topological transformations that occur during the reaction process involved in molecular decomposition [36]. The study aimed to develop and apply a quantum theory of atoms in molecules for evaluating the susceptibility of bonds to cleavage in four highly hazardous pesticides. It was also relevant to clarify their molecular reactivity profiles and identify electronically labile pertinent bonds to their environmental degradation, toxicological activation, and risk assessment. In recent years, global and local DFT reactivity descriptors have proven to be useful tools for evaluating the biodegradability and toxicity of organic molecules [37,38].

## 2. Materials and Methods

### 2.1. Pesticide Classification

Four organophosphate insecticides were chosen based on their toxicity and known effects on human health and the environment. Selection was made using entries from the University of Hertfordshire’s Pesticide Properties DataBase (PPDB) [39] and WHO hazard classification criteria [16]. For each insecticide, we include data such as CAS, RN, IUPAC name, key physicochemical properties, and mammalian acute toxicity endpoints. WHO hazard categories are primarily assigned from acute oral LD 50 values, classifying them as extremely hazardous (Ia), highly hazardous (Ib), moderately hazardous (II), or slightly hazardous (III) [16].

Diazinon CAS RN: 333–415; IUPAC: O, O-Diethyl O-(2-isopropyl-6-methylpyrimidin-4-yl) phosphorothioate; water solubility of 60 mg/L^−1^ at 20 °C; log P of 3.69 at pH 7 and 20 °C; vapor pressure of 11. 97 mPa at 20 °C; Henry’s constant of 6.09 × 10^−2^ Pa·m^3^/mol^−^; and pKa of 2.6. Its acute toxicity in rats includes an oral LD_50_ of 1139 mg/kg^−1^, dermal LD_50_ of 2000 mg/kg^−1^, and inhalation LC_50_ greater than 5.0 mg/L^−1^. It is classified as WHO hazard class II (Moderately hazardous).

Chlorpyrifos CAS RN: 2921-88-2; IUPAC: O,O-Diethyl O-(3,5,6-trichloro-2-pyridyl) phosphorothioate; water solubility of 1.05 mg/L^−1^ at 20 °C; log P of 4.7 at pH 7 and 20 °C; vapor pressure of 2.5 mPa at 20 °C. Its acute rat toxicity includes an oral LD_50_ of 66 mg/kg^−1^, dermal LD_50_ of 1250 mg/kg^−1^, and inhalation LC_50_ of 0.1 mg/L^−1^. It is also classified as WHO hazard class II (Moderately hazardous).

Acephate CAS RN: 30560-19-1; IUPAC: N-acetyl-O,S-Dimethylphosphoramidothioate; water solubility of 650,000 mg/L^−1^ at 20 °C; log P of −0.85 at pH 7 and 20 °C; vapor pressure of 0.0072 mPa at 20 °C. Its acute toxicity includes an oral LD_50_ of 945 mg/kg^−1^, dermal LD_50_ of 10,000 mg/kg^−1^, and inhalation LC_50_ of 15 mg/L^−1^, placing it in WHO hazard class II (Moderately hazardous).

Methamidophos CAS RN: 10265-92-6; IUPAC: O,S-Dimethyl phosphoramidothioate; water solubility is 200,000 mg/L^−1^ at 20 °C; log P of −0.79 at pH 7 and 20 °C; vapor pressure of 2.3 mPa at 20 °C; Henry’ s constant of 1.60 × 10^−6^ Pa·m^3^/mol^−1^. In rats, it has an oral LD_50_ of 20 mg/kg^−1^, dermal LD_50_ of 130 mg/kg^−1^, and inhalation LC_50_ of 1.37 mg/L^−^1. It is classified as WHO hazard class Ib (Highly hazardous).

### 2.2. Computational Details

**Figure 1:**
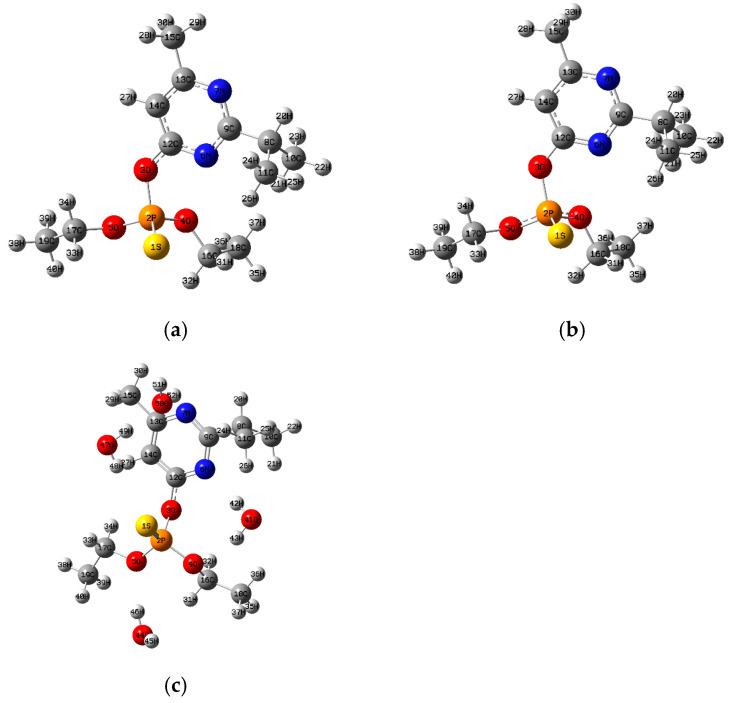
(**a**) Diazinon molecule optimized in the gas phase, (**b**) Diazinon molecule optimized in water as dielectric continuum, (**c**) Diazinon supermolecule surrounded by four water molecules optimized in water as dielectric continuum.

**Figure 2:**
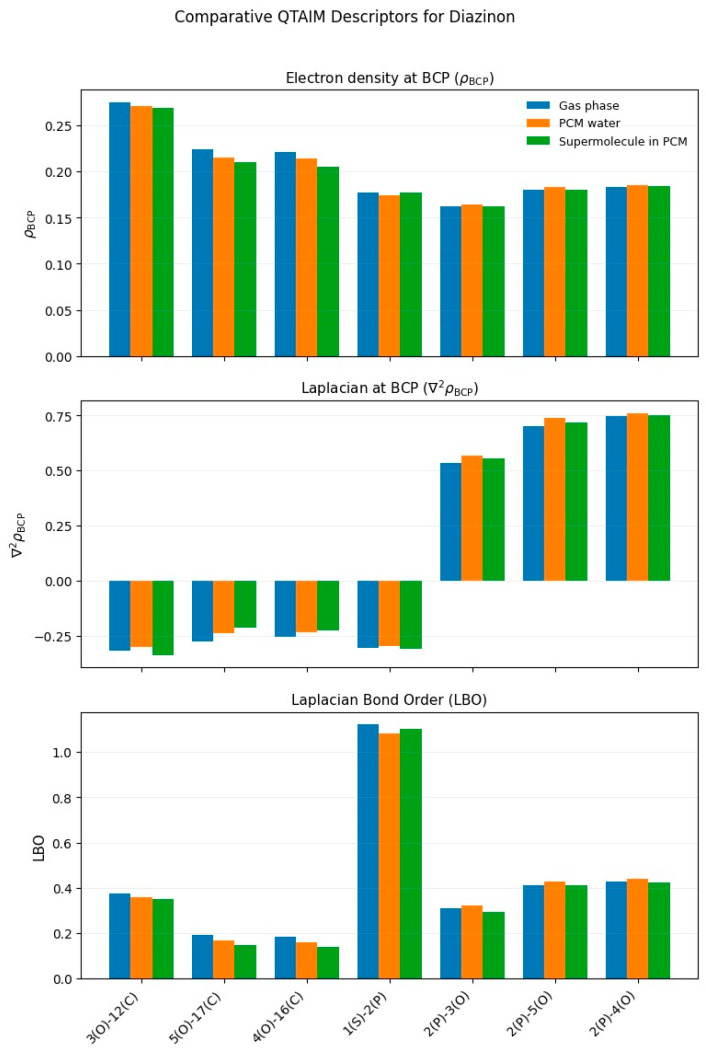
Comparative QTAIM descriptors for Diazinon in gas phase, PCM water, and the supermolecule in PCM (water as continuum). The plots show (**top**) electron density at the bond critical point (ρ BCP), (**middle**) Laplacian of the electron density (∇^2^ρ BCP), and (**bottom**) Laplacian Bond Order (LBO) for the bonds indicated on the *x*-axis.

**Figure 3:**
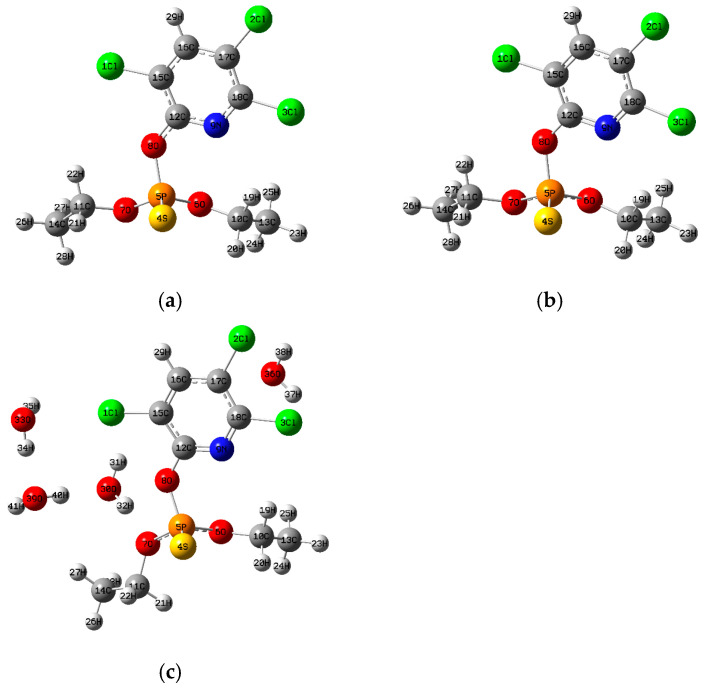
(**a**) Chlorpyrifos molecule optimized in the gas phase, (**b**) Chlorpyrifos molecule optimized in water as dielectric continuum, (**c**) Chlorpyrifos supermolecule surrounded by four water molecules optimized in water as dielectric continuum.

**Figure 4:**
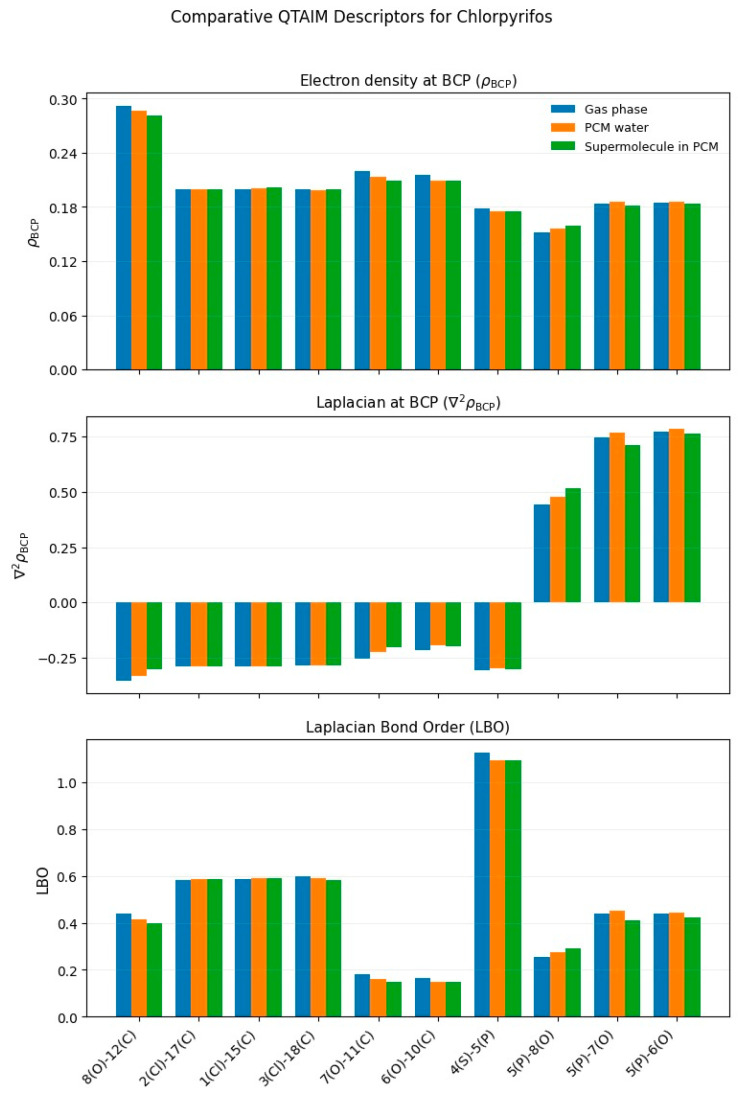
Comparative QTAIM descriptors for Chlorpyrifos in gas phase, PCM water, and the supermolecule in PCM (water as continuum). The plots show (**top**) electron density at the bond critical point (ρ BCP), (**middle**) Laplacian of the electron density (∇^2^ρ BCP), and (**bottom**) Laplacian Bond Order (LBO) for the bonds indicated on the *x*-axis.

**Figure 5:**
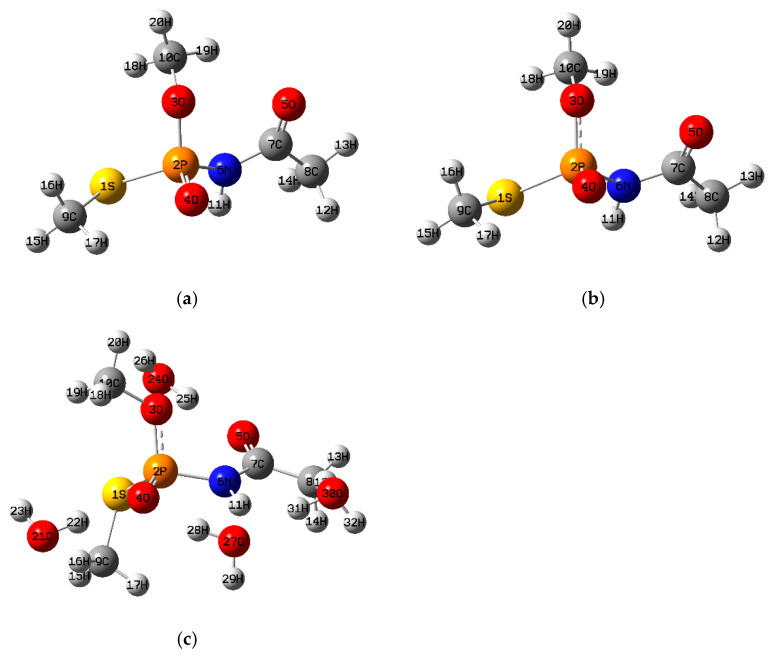
(**a**) Acephate molecule optimized in the gas phase, (**b**) Acephate molecule optimized in water as dielectric continuum, (**c**) Acephate supermolecule surrounded by four water molecules optimized in water as dielectric continuum.

**Figure 6:**
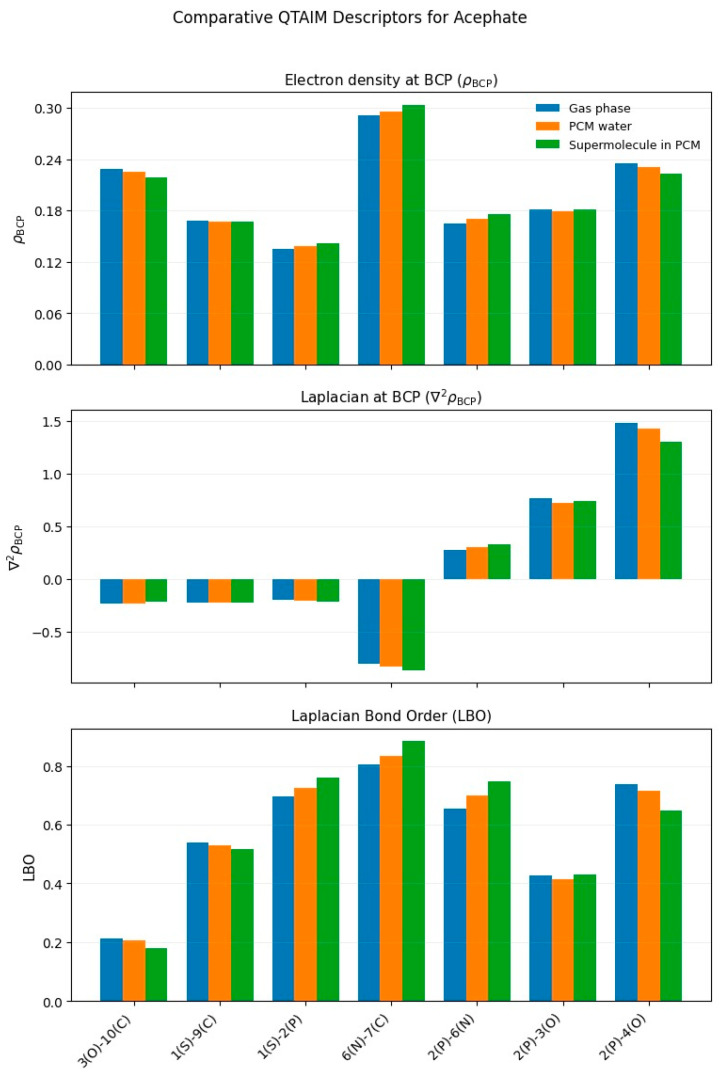
Comparative QTAIM descriptors for Acephate in gas phase, PCM water, and the supermolecule in PCM (water as continuum). The plots show (**top**) electron density at the bond critical point (ρ BCP), (**middle**) Laplacian of the electron density (∇^2^ρ BCP), and (**bottom**) Laplacian Bond Order (LBO) for the bonds indicated on the *x*-axis.

**Figure 7:**
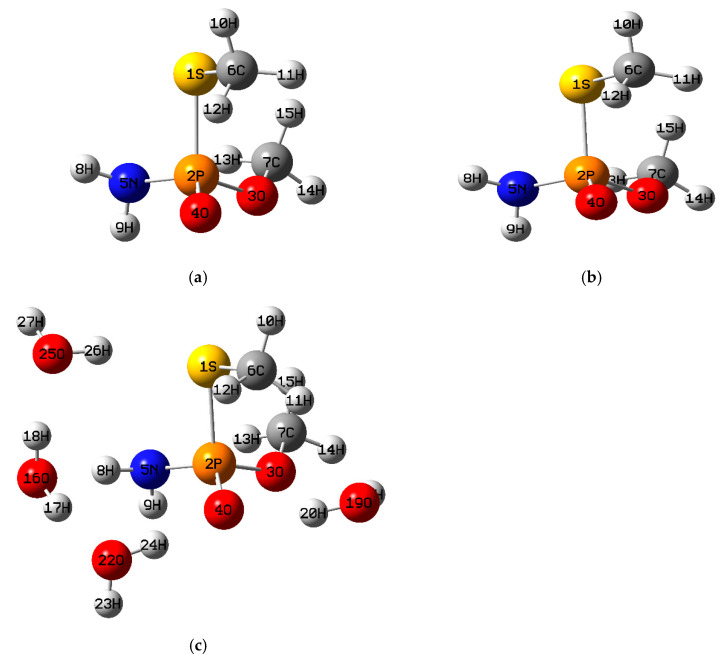
(**a**) Methamidophos molecule optimized in the gas phase, (**b**) Methamidophos molecule optimized in water as dielectric continuum, (**c**) Methamidophos supermolecule surrounded by four water molecules optimized in water as dielectric continuum.

**Figure 8:**
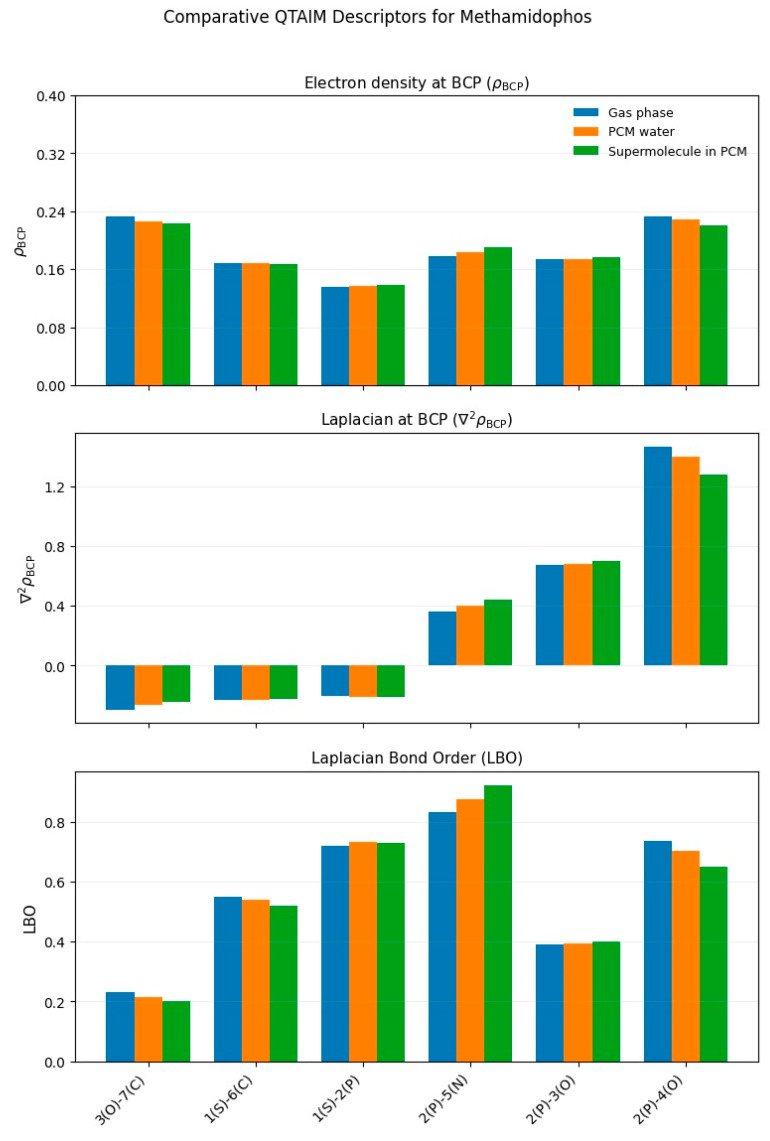
Comparative QTAIM descriptors for methamidophos in gas phase, PCM water, and the supermolecule in PCM (water as continuum). The plots show (**top**) electron density at the bond critical point (ρ BCP), (**middle**) Laplacian of the electron density (∇^2^ρ BCP), and (**bottom**) Laplacian Bond Order (LBO) for the bonds indicated on the *x*-axis.

The theoretical calculations were carried out with the Gaussian 16 program [40]. The optimized structures of the four organophosphate pesticides and their corresponding topological graphs are shown in Figure 1, Figure 2, Figure 3, Figure 4, Figure 5, Figure 6, Figure 7 and Figure 8: diazinon (Figure 1a and Figure 2), chlorpyrifos (Figure 3a and Figure 4), acephate (Figure 5a and Figure 6), and methamidophos (Figure 7a and Figure 8). All molecular geometries were optimized in the gas phase at the DFT level using the B3LYP functional and the 6–311G(d,p) basis set (Figure 3a). 

Topological bond analyses of the molecules were carried out using the QTAIM method with Multiwfn 3.8 software [41]. To assess the likelihood of bond rupture, QTAIM topological descriptors such as Laplacian bond order [42], Bond Order Analysis Based on the Laplacian of Electron Density in Fuzzy Overlap Space [43], the electron density value, and the Laplacian of the electron density at the bond critical point were analyzed [43].

Bonds susceptible to breaking were identified as having values of either a low Laplacian bond order (LBO < 1), a small electron density value at the bond critical point (ρ BCP) regarding the total bond values in the pesticide molecule, or a positive value for the Laplacian of the electron density at the BCP (∇^2^ρ BCP > 0). To evaluate solvent effects, all four pesticides were also optimized in water using the PCM (Polarizable Continuum Model) [44]. The optimized molecular structures and their corresponding topological descriptor graphs are shown in: diazinon (Figure 1b and Figure 2), chlorpyrifos (Figure 3b and Figure 4), acephate (Figure 5b and Figure 6), and methamidophos (Figure 7b and Figure 8).

In addition, supermolecular models including four explicit water molecules within the PCM framework were generated for each pesticide. The optimized molecular structures and their corresponding topological descriptor graphs are presented in: diazinon (Figure 1c and Figure 2), chlorpyrifos (Figure 3c and Figure 4), acephate (Figure 5c and Figure 6), and methamidophos (Figure 7c and Figure 8). The water molecules were initially placed in random positions around each pesticide and subsequently optimized together with the solute. Post-optimization NCI plot analyses were conducted using Multiwfn 3.8 [41] in the absence of previous molecular dynamics simulations. No index between the geometries of each pesticide in the gas phase, PCM water, and the supermolecule in PCM was computed. The QTAIM analysis was complemented with global reactivity descriptors such as chemical potential (μ), hardness (η), and electrophilicity (ω) as well as local reactivity descriptors, namely the condensed Fukui functions (*f***^+^**), calculated with the AOMIX software version 6.6 [45].

The topological analysis of the Fukui function (TAFF) was performed to characterize local reactivity patterns of the studied pesticides. This method combines the computation of Fukui functions with a topological partition of the electron density into QTAIM basins, allowing the identification of nucleophilic (*f*^+^), electrophilic (*f*^−^), and radical (*f*^0^) attractors. Calculations were carried out with the TAFF software version 1.0 [46], a C++ pipeline that applies the Koopmans and frozen orbital approximations, where *f*^+^ is obtained from the squared LUMO, *f*—from the squared HOMO, and *f*^0^ from their sum. The procedure integrates the resulting functions within each atomic basin by automatic calls to Multiwfn [41], providing spatially resolved indices that map reactivity descriptors directly onto the QTAIM topology.

## 3. Results and Discussion

### 3.1. Topological Analyses of Thiophosphate Molecules

#### 3.1.1. Diazinon

In the QTAIM topological analysis of diazinon in the gas phase (Figure 2), three P–O bonds (Figure 1a–c) were identified (P(2)–O(3), P(2)–O(4), and P(2)–O(5)), whose characterization is key to understanding both the molecular stability and its biological reactivity. All P–O bonds display reduced electron density at the bond critical points (ρ BCP 0.162–0.183 a.u.) compared to C–N, C–C, and C–H bonds within the same molecule (see Support Information, Table S1). They also exhibit positive Laplacian values (∇^2^ρ BCP = 0.535–0.747 a.u.) and Laplacian Bond Order (LBO) values below 1. Single P–O bonds such as P(2)–O (4) and P(2)–O (5) show high susceptibility to polarization and possible hydrolysis. However, experimental evidence indicates that the P(2)–O(3) bond is the preferential site for hydrolysis [47,48] and is also susceptible to enzymatic [49] and microbial degradation [50]. The principal differences in the lability of the P–O bonds in diazinon arise from substituent effects and electronic polarization. The P(2)–O(3) bond is attached to the electron-withdrawing pyrimidine ring, which promotes bond polarization, resulting in a lower electron density at the BCP and a reduced Laplacian bond order compared to the P(2)–O(4) and P(2)–O(5) bonds. In contrast, the latter are connected to ethyl substituents (–C_2_H_5_) with electron-releasing character, which induce less polarization.

Additionally, the P–S bond (P(1)–S(1), Figure 1a–c) exhibits a particular behavior. Although the LBO value (1.12) together with a negative Laplacian at the BCP (∇^2^ρ BCP = −0.306) indicates a significant covalent character, the electron density at the BCP (ρ BCP = 0.17) is relatively low compared to typical covalent bonds. This finding suggests that, although the bond has a covalent structure, its reduced electron density makes it prone to cleavage, supporting its role as the primary reactive site during oxidation to diazoxon [51]. This more reactive metabolite retains the phosphoryl group (P=O) and exhibits higher toxicity. Therefore, the QTAIM characterization supports the idea that the phosphorylated environment of diazinon, notably the P–O and P–S bonds, contains chemically labile regions that are critical to its activation mechanism and toxicity.

On the other hand, the C–O bond of the ether group (O(3)–C(12)) and the O(5)–C(17) and O(4)–C(16) bonds (Figure 1a–c), corresponding to alkoxy substituents in the phosphorylated environment, tend to reduced electron density. These bonds show reduced values of electron density at the BCP (ρ BCP) and LBO, indicative of weak covalency, although their Laplacian values remain negative. This topology is consistent with the expected chemical behavior, particularly for the O(3)–C(12) bond, whose cleavage is a well-established degradation pathway [52,53]. Therefore, although the C–O bonds are not primary reactive centers like the P–S or P–O linkages, their electronic profile suggests that they may act as secondary sites prone to cleavage.

The influence of a dielectric continuum model such as PCM with water as the solvent, and the formation of a supermolecule including four explicit water molecules surrounding the structure along with PCM, did not significantly affect the topological parameters (Figure 2). Only slight increases were observed in the Laplacian at the BCP values for the P–O bonds, along with slight decreases in the Laplacian Bond Order values for the C–O bonds and for the P(2)–O(3) bond in the supermolecular model. Nevertheless, the overall trend remains consistent with the topological analysis carried out in the gas phase.

#### 3.1.2. Chlorpyriphos

Another thiophosphorylated molecule analyzed topologically in the gas phase through QTAIM is chlorpyrifos (Figure 4). Like diazinon, three P–O bonds were identified, exhibiting reduced electron density values at the bond critical points (ρ BCP = 0.152–0.185 a.u.) comparable to those of diazinon, as well as positive Laplacian values (∇^2^ρ BCP = 0.442–0.774 a.u.). These findings are consistent with experimental evidence, since the P(5)–O(8) bond (Figure 3a–c) is the most susceptible to hydrolysis [54]. When considering substituent effects, a similar trend to that observed in diazinon is found in chlorpyrifos, where the P–O bond connected to the trichlorinated pyridine ring displays stronger polarization than the P–O bonds linked to alkyl substituents. The pronounced electron-withdrawing character of the halogenated pyridine increases bond polarization, reducing the electron density at the BCP and the Laplacian bond order, which explains its higher lability relative to the alkyl-bound P–O bonds. Regarding the P(5)–S(4) bond (Figure 3a–c), its covalency is supported by a negative Laplacian value at the BCP (∇^2^ρ BCP = −0.307) and a relatively high Laplacian Bond Order (LBO = 1.13).

Nevertheless, the electron density at the BCP (ρBCP = 0.17) is considerably lower than that found in typical covalent bonds such as C–N, C–C, and C–H (see Supporting Information Table S4). This suggests that, despite its covalent structure, the P–S bond displays reduced electron density, making it susceptible to oxidation into chlorpyrifos-oxon. This product retains the phosphoryl group (P=O) and exhibits higher toxicity [55,56,57].

In this molecule, three C–O bonds (O(8)–C(12), O(6)–C(10), and O(7)–C(11), Figure 3a–c) were also found to exhibit features like those in diazinon. All three display negative Laplacian values (∇^2^ρ BCP = −0.215 to −0.353), indicating covalent character at first glance. However, their electron density values at the BCP are relatively low (ρ BCP = 0.215–0.292 a.u.) and their LBO values are below 1 (LBO = 0.166–0.439), which reveals the lability of these C–O bonds. Dealkylation of O(6)–C(10) and O(7)–C(11) has been reported in later stages when chlorpyrifos undergoes thermoactivated persulfate oxidation [56].

The three C–Cl bonds (Cl(1)–C(15), Cl(2)–C(17), and Cl(3)–C(18), Figure 3a–c) show negative Laplacian values at the BCP (∇^2^ρ BCP = −0.287 to −0.290), which is indicative of their covalent nature. However, their electron density values (ρ BCP ≈ 0.200 a.u.) are relatively low compared with those of C–N, C–C, and C–H bonds (Table S4), and their LBO values are moderate (0.584–0.598). A proposed reaction pathway for the oxidation of chlorpyrifos using Fe(VI) [23] highlights degradation through hydroxyl substitution reactions. Although no primary degradation mechanisms involve direct cleavage of the C–Cl bonds, several studies have demonstrated that dichlorination can occur under photodegradation [24,58], biodegradation [59], or thermoactivated persulfate oxidation [56], supporting the possibility of C–Cl bond rupture at later stages. Calculations in PCM with water as dielectric and in the supermolecular model (Figure 4), including explicit water molecules, showed an increase in the Laplacian values at the BCP for the hydrolysis-susceptible P(5)–O(8) bond, together with decreases in the LBO values for the C–O bonds. From the gas phase to PCM and supermolecular conditions, no significant variations were observed in the remaining topological descriptors for the studied bonds.

#### 3.1.3. Acephate

The QTAIM-based topological analysis of acephate in the gas phase (Figure 6) revealed only two P–O bonds (P(2)–O(3) and P(2)–O(4), Figure 5a–c), both of which exhibit reduced electron density values at the bond critical points (ρ BCP = 0.181, 0.235 a.u., respectively) compared with those observed for N–C, C–C, C–H, and O–C bonds (e.g., O(5)–C(7) carbonyl, Table S7 in Support Information). These P–O bonds also show positive Laplacian values at the BCP (∇^2^ρ BCP = 0.769, 1.486 a.u.) and LBO values below 1. A theoretical study on the hydrolysis mechanism of acephate proposed both a concerted and a stepwise pathway involving these bonds [30].

The P–N bond (P(2)–N(6), Figure 5a–c) follows the same trends as the P–O bonds in terms of topological descriptors (ρ BCP = 0.165 a.u., ∇^2^ρ BCP = 0.271 a.u., LBO = 0.271). The cleavage of this bond is known to yield a derivative of acephate called O,S-dimethyl O-hydrogen phosphorothioate (O,S-DMPT), formed through hydrolysis [60], and microbial degradation of acephate [61]. Although the N–C bond (N(6)–C(7), Figure 5a–c) has an LBO value below 1, it shows a relatively high electron density at the BCP (ρ BCP = 0.291 a.u.), comparable to that of C–H bonds in the molecule (Table S7), and a negative Laplacian (∇^2^ρ BCP = −0.809 a.u.), consistent with covalent character. However, it is well known that the rupture of this N–C bond leads to the formation of methamidophos, a product of acephate biodegradation [62,63]. The S–P bond (S(1)–P(2), Figure 5a–c) presents an LBO value below 1 and the lowest electron density at the BCP among all bonds in the molecule (ρ BCP = 0.135 a.u.), together with a negative Laplacian value (∇^2^ρ BCP = −0.198 a.u.). The hydrolysis of this bond has been theoretically studied [27], and experimental results indicate that it can be cleaved under alkaline hydrolysis at pH = 11 [29] and by biodegradation [62]. Although the S–C bond (S(1)–C(9), Figure 5a–c) and the O–C bond (O(3)–C(10), Figure 5a–c) display similar topological descriptor values to S–P, no primary bond cleavages involving these linkages have been yet reported in the literature.

The use of water as a dielectric continuum (PCM) and the supermolecular model (Figure 6) with explicit water molecules did not modify the <0 or >0 trends of the Laplacian values at the BCP compared with the gas phase. No significant changes were observed in the electron density at the BCP either. On the other hand, LBO values remained below 1, but with slight tendencies to increase from gas phase → PCM → supermolecule PCM (S(1)–P(2), N(6)–C(7), and P(2)–N(6)) and to decrease in the same sequence for O(3)–C(10), S(1)–C(9), and P(2)–O(4).

#### 3.1.4. Methamidophos

Methamidophos can be obtained from acephate [63]. QTAIM-based topological analysis of methamidophos was carried out in the optimized molecule from the gas phase (Figure 8). Similar to its parent compound acephate, it presents two P–O bonds (P(2)–O(3) and P(2)–O(4), Figure 7a–c), whose electron density values at the bond critical points (ρ BCP = 0.174, 0.233 a.u., respectively) are markedly lower than those found for N–C, N–H, and C–H bonds (see Support Information, Table S10). The Laplacian at the BCP and LBO values follow the same trend as in acephate: positive ∇^2^ρ BCP values (0.674, 1.466 a.u.) and LBO values below 1. Experimentally, cleavage of the P(2)–O(3) bond is favored, through which methamidophos is presumably converted into S-methyl O-hydrogen phosphorothioamidate by biodegradation in M9 medium [64].

Hydrolysis at pH = 2 [29], and biodegradation of methamidophos also yield O,S-DMPT [59,61]. This occurs mainly through the cleavage of the P–N bond (P(2)–N(5), Figure 7a–c), whose topological descriptor values support this reactivity (ρ BCP = 0.178 a.u., ∇^2^ρ BCP = 0.362 a.u., LBO = 0.832).

The LBO value below 1 and the lowest electron density at the BCP in the entire molecule (ρ BCP = 0.136 a.u.) for the S–P bond (S(1)–P(2), Figure 7a–c) suggest its lability, considering that, as in acephate, the Laplacian value at the BCP is negative. Experimentally, this bond undergoes hydrolysis under basic conditions at pH = 11, producing O-methyl phosphoramidate [29,64,65,66,67]. In comparison with acephate, the S–C bond (S(1)–C(6)) and the O–C bond (O(3)–C(7), Figure 7a–c) also exhibit similar topological descriptor values to S–P, with no reports of homolytic or heterolytic cleavage in early degradation stages.

The use of water as a dielectric continuum, as well as the inclusion of the supermolecular model, did not alter the <0 or >0 trends of the Laplacian values at the BCP observed in the gas phase (Figure 8). Similarly, no significant changes were recorded in the electron density at the BCP. On the other hand, LBO values remained below 1 in all cases, although with certain variations across the sequence gas phase → PCM → supermolecule. Specifically, the P(2)–N(5) bond showed a tendency toward increasing LBO values, whereas the P(2)–O(4) bond exhibited decreasing values in this sequence.

### 3.2. Comparative Analysis Across Pesticides

A comparative examination of the four organophosphate pesticides highlights common and divergent features in their topological profiles. In all cases, P–O bonds display reduced electron density at the BCP and LBO values below 1, indicating their intrinsic lability regardless of the solvation model. Nevertheless, substituent effects modulate their relative weakness: in diazinon and chlorpyrifos, the P–O bond attached to the heteroaromatic ring (pyrimidine or trichlorinated pyridine, respectively) exhibits greater polarization than those linked to alkyl substituents. In acephate and methamidophos, the P–N bond emerges as an additional cleavage-prone site, consistent with known hydrolytic and biodegradation pathways. Across all systems, the P–S bond also shows relatively low electron density values compared with typical covalent linkages, reinforcing its role as a key reactive center during oxidative transformations. Importantly, the solvent models (PCM water and supermolecule in PCM) produced only slight variations in the descriptors, confirming that these QTAIM parameters are largely intrinsic to the electronic structure of each pesticide. This comparative perspective complements the molecule-by-molecule discussion and provides a unified view of how structural motifs influence bond lability and degradation routes.

## 4. Global and Local Reactivity Descriptors

In addition to the topological analysis provided by QTAIM, which identifies intrinsic bond properties through parameters such as electron density (ρ BCP), Laplacian (∇^2^ρ BCP), and Laplacian bond order (LBO), we complemented our study with global and local reactivity descriptors. First, the dipole moment and total energy of the studied systems vary significantly across the gas phase, PCM, and supermolecule in PCM models (Table S14). Global reactivity indices [37,38], including chemical potential (μ), hardness (η), and electrophilicity (ω), offer a quantitative measure of the overall reactivity patterns of the pesticides and their variation with solvation. Local reactivity descriptors, namely the condensed Fukui functions (*f*^+^) [38], provide atom specific insight into the susceptibility of different sites toward nucleophilic attack. The combination of QTAIM descriptors with conceptual DFT indices thus enables a more comprehensive evaluation of bond lability and solvent effects on electronic structure and reactivity.

### 4.1. Influence of Solvation on Frontier Molecular Orbitals and Global Reactivity Descriptors

The inclusion of solvation effects, either through PCM water or by explicit water molecules in the supermolecule in PCM model, induces systematic changes in the frontier orbital energies of the pesticides compared to the gas phase. In general, the HOMO levels experience a slight stabilization (more negative values) in solution, while the LUMO levels also shift to lower energies, particularly under PCM conditions. This combined effect results in moderate variations in the HOMO–LUMO gap, which are directly reflected in the global descriptors.

For hardness (η), all molecules display slightly higher values in solution compared with the gas phase, with methamidophos consistently showing the largest η values, confirming its greater electronic stability. The chemical potential (μ) also becomes more negative upon solvation, reflecting the stabilization of the electronic density distribution in a polar medium. Electrophilicity (ω) exhibits more molecule-dependent trends: chlorpyrifos shows the highest values in both PCM and supermolecule models, consistent with the strong electron-withdrawing effect of its trichlorinated pyridine ring, while diazinon presents the second-highest ω value due to the polarization induced by its pyrimidine ring. Acephate and methamidophos display comparatively lower ω values.

**Figure 9:**
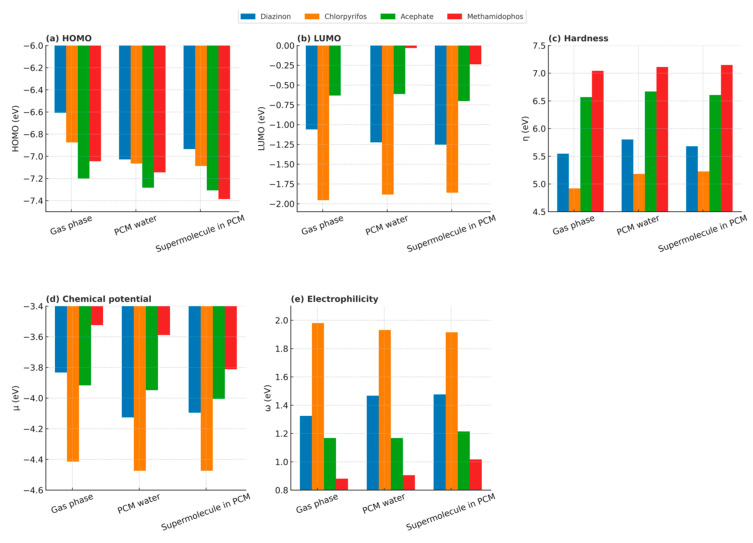
Frontier orbital energies and global reactivity descriptors of Diazinon, Chlorpyrifos, Acephate, and Methamidophos in different solvation environments. Subpanels show (**a**) HOMO energies, (**b**) LUMO energies, (**c**) hardness (η), (**d**) chemical potential (μ), and (**e**) electrophilicity (ω). Results are presented for gas phase, PCM water, and supermolecule in PCM conditions. The comparison highlights the influence of solvation on electronic structure and global reactivity descriptors, providing insights into the relative stability and potential reactivity of each pesticide.

It is also noteworthy that, for acephate and methamidophos, the PCM water model produces only minor variations relative to the gas phase, whereas the supermolecule in PCM approach yields more pronounced changes in the descriptors (Figure 9). This indicates that explicit hydrogen-bond interactions with water molecules can significantly influence electronic reactivity patterns, beyond the continuum polarization captured by PCM.

Overall, the results show that solvation slightly stabilizes frontier orbitals and enhances global descriptors of reactivity without significantly altering the relative trends among the four pesticides. The similarity between PCM water and the supermolecule in PCM approach in chlorpyrifos, together with the larger differences in the other three pesticides, highlights the interplay between continuum polarization and explicit hydrogen bonding in modulating pesticide reactivity. Further, the NCI plot for chlorpyrifos displays a markedly sparser blue/green region at negative sign (λ_2_)ρ, indicating fewer and weaker hydrogen bond interactions with the aqueous environment (Figure S14).

### 4.2. The Condensed Electrophilic Fukui Functions (f^+^) and Solvent Effects

**Figure 10:**
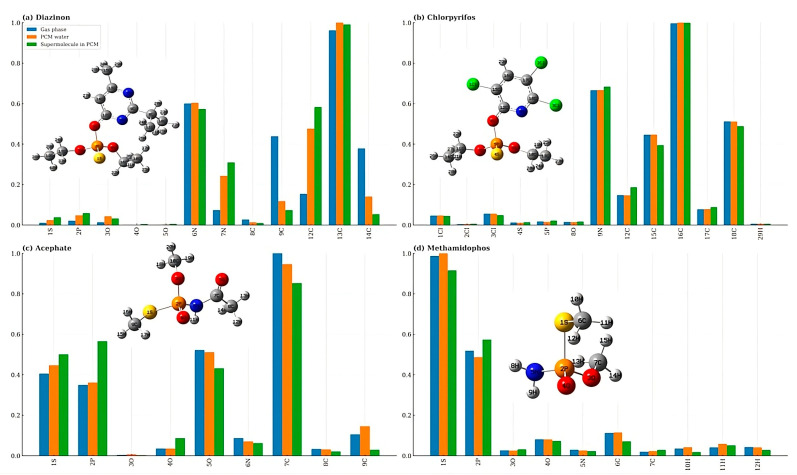
Normalized condensed Fukui *f*^+^ functions (0–1) for (**a**) Diazinon, (**b**) Chlorpyrifos, (**c**) Acephate, and (**d**) Methamidophos under three conditions: gas phase, PCM water, and supermolecule in PCM. Bars represent atom-specific electrophilic reactivity indices, highlighting the relative susceptibility of different atomic sites toward nucleophilic attack. The consistent normalization across molecules facilitates direct comparison of reactive sites and the effect of solvation environment on electrophilic reactivity.

This kind of Fukui function reveals the most electrophilically susceptible sites in each pesticide and allows the evaluation of solvent effects on local reactivity. For diazinon (Figure 10a), the nitrogen atoms and the aromatic carbons of the pyrimidine ring display the highest *f*^+^ values, with a noticeable increase under the supermolecule in PCM model. This enhanced susceptibility is consistent with the polarization of the P–O bond connected to the heteroaromatic ring, previously identified as a labile linkage in the QTAIM analysis.

In chlorpyrifos (Figure 10b), the nitrogen atom and selected carbons of the trichlorinated pyridine ring concentrate the highest *f*^+^ values. These sites exhibit relatively minor changes between gas phase and PCM water, whereas the supermolecule in PCM model shows only a slight amplification of the local reactivity at the pyridine carbons, consistent with the limited hydrogen-bonding interactions observed between water molecules and the aromatic ring. This trend parallels the QTAIM finding that the P–O bond linked to the halogenated aromatic ring is more polarized and therefore more labile than the alkyl-bound P–O bonds.

In acephate (Figure 10c), the condensed electrophilic Fukui functions highlight C(7), P(2) and S(1) as the most susceptible sites. This pattern is fully consistent with the experimentally and mechanistically relevant cleavage channels S(1)–P(2), P(2)–N(6), and N(6)–C(7) (en route to methamidophos). The large *f*^+^at P(2) indicates an electron-accepting center prone to nucleophilic attack at phosphorus, which weakens both the S–P and P–N bonds and rationalizes their observed lability in the QTAIM analysis (lower ρBCP and reduced LBO). The high *f*^+^at S(1) reflects polarization of the S-P fragment, further facilitating S–P scission under nucleophilic conditions. Likewise, the prominent *f*^+^at C(7) supports N–C cleavage (demethylation) by indicating an electrophilic carbon adjacent to the amide nitrogen. Solvation modulates these trends only modestly in PCM water, whereas the supermolecule in PCM produces a clearer amplification of *f*^+^at P(2) and S(1), reinforcing the propensity of the S–P and P–N bonds to rupture in an aqueous environment.

For methamidophos (Figure 10d), the condensed electrophilic Fukui functions identify P(2) and S(1) as the dominant electron-accepting sites (highest *f* values), in line with the experimentally relevant scission channels P(2)–O(3), P(2)–N(5), and S(1)–P(2). A large *f*^+^ at P(2) indicates that nucleophilic attack at phosphorus will preferentially weaken the P–O and P–N linkages, while the enhanced *f*^+^ at S(1) reflects polarization of the thionyl fragment and facilitates S–P cleavage. These Fukui trends are consistent with QTAIM indicators of reduced bond stability at the same bonds. Solvation perturbs local reactivity, resulting in a modest change in PCM water. However, the supermolecule in PCM amplifies *f*^+^ at P(2) and S(1), thereby enhancing the propensity for P–O, P–N and S–P cleavage in aqueous environments.

### 4.3. Topological Analysis of the Fukui Function (TAFF)

To further integrate the QTAIM framework with local reactivity descriptors, we performed a Topological Analysis of the Fukui Function (TAFF) [68]. This approach partitions the Fukui functions into QTAIM basins, allowing the direct mapping of nucleophilic (*f***^−^**), electrophilic (*f***^+^**), and radical (*f*^0^) susceptibilities onto the same topological space used for bond critical point analysis. The TAFF calculations were carried out for all four pesticides in the gas phase, since, as shown in our QTAIM results, the topological features remain largely unchanged under solvation.

**Figure 11:**
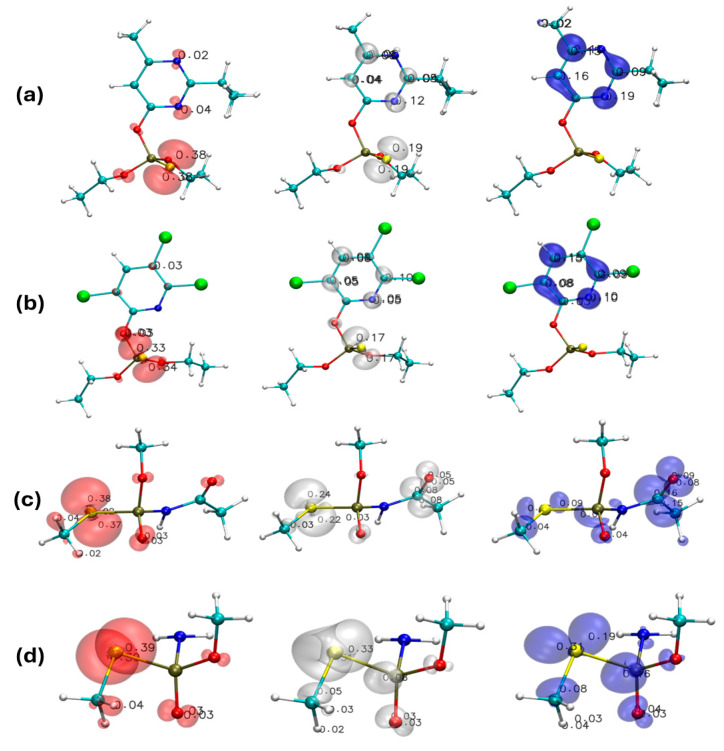
Isosurface representation (isovalue 0.005 a.u.) of the Fukui functions obtained from the TAFF (Topological Analysis of the Fukui Function) for the four pesticides studied: (**a**) Diazinon, (**b**) Chlorpyrifos, (**c**) Acephate, and (**d**) Methamidophos. Red lobes correspond to the nucleophilic Fukui function (*f*^−^), blue lobes to the electrophilic Fukui function (*f*^+^), and grey lobes to the radical Fukui function (*f*^0^).

For diazinon (Figure 11a), the *f*—function localized on the sulfur atom and the nitrogen atoms of the pyrimidine ring, reflecting their electron-rich nature and consistency with QTAIM descriptors that indicated low ρ BCP and LBO for P–S and P–O linkages. The *f*^+^ attractors concentrated on the pyrimidine ring, supporting its role as an electron-deficient region polarized by the heteroaromatic substituent. The *f*^0^ distribution, spanning both sulfur and the aromatic ring, indicates radical susceptibility at the same centers, thus reinforcing their mechanistic relevance in oxidative and biodegradative pathways.

In chlorpyrifos (Figure 11b), the *f*—density was concentrated almost exclusively on the sulfur atom, consistent with QTAIM characterization of the P–S bond as covalent but with reduced electron density, making it prone to oxidative activation into chlorpyrifos-oxon. Conversely, *f*^+^ values were localized on the trichloropyridine ring, highlighting the electron-deficient character of this substituent and rationalizing the preferential hydrolysis of the adjacent P–O bond. The *f*^0^ pattern exhibited a bimodal distribution over sulfur and the aromatic ring, further supporting their dual role as reactive sites for radical processes.

For acephate (Figure 11c), the TAFF analysis revealed significant *f*^+^ values at phosphorus, sulfur, and the carbon atom from the carbonyl group, which coincide with the experimentally relevant cleavage channels S–P, P–N, and N–C (in the pathway to methamidophos). These findings are fully consistent with QTAIM descriptors that identified these bonds as weak (low ρ BCP, LBO < 1). The high *f*—and *f*^0^ values at sulfur further reinforce its role as an electron rich center, polarizing the S–P bond and enhancing its susceptibility to hydrolysis, in agreement with the trends previously observed in the topological analysis.

Finally, methamidophos (Figure 11d) exhibited a broader reactivity pattern. The *f*—function was concentrated on sulfur and terminal oxygen atoms, while *f*^+^ displayed an extended distribution including sulfur, phosphorus, and terminal oxygen. This wider spread of nucleophilic susceptibility mirrors the QTAIM observation that several bonds (P–O, P–N, S–P) exhibit low electron density and reduced bond order. The overlap of *f*^0^ with *f*^+^ at these same sites suggests that electrophilic and radical reactivity converge on the same centers, rationalizing the high hydrolytic lability and multiple experimentally reported degradation routes of methamidophos.

Overall, the integration of TAFF with QTAIM and conceptual DFT descriptors provides a coherent mechanistic framework. QTAIM identifies electronically weak bonds through topological parameters (ρ BCP, ∇^2^ρ BCP, LBO), global and local DFT descriptors reveal solvent modulation of reactivity, and TAFF bridges both by mapping nucleophilic, electrophilic, and radical susceptibilities directly onto the QTAIM topology. This combined approach confirms that the cleavage-prone bonds identified in our study are not only intrinsic electronic weak points but also preferential sites for environmental degradation pathways.

## 5. Conclusions

Understanding the bond rupture pathways of Highly Hazardous Pesticides is fundamental due to their direct implications for human health and environmental safety. The QTAIM analysis proved highly effective in identifying electronically weak and chemically unstable bonds, notably P–O and P–S linkages across all pesticides, and P–N bonds in acephate and methamidophos. These results provide a consistent theoretical rationale for their known hydrolytic, oxidative, microbial, and enzymatic degradation pathways.

A comparative perspective confirmed that QTAIM descriptors are essentially intrinsic to the molecular electronic structure. Neither the dielectric continuum (PCM) nor the explicit solvation (supermolecule in PCM) produced significant changes in electron density, Laplacian, or Laplacian Bond Order trends, demonstrating that bond topology remains largely independent of the medium.

In contrast, analyzed molecular orbital energy levels, global and local reactivity descriptors (HOMO, LUMO, hardness, chemical potential, electrophilicity, and condensed Fukui functions) showed more pronounced sensitivity to solvation. These indices highlight subtle but relevant shifts in frontier orbital energies, global stability, and site-specific susceptibility, particularly under explicit solvent interactions. Thus, while QTAIM provides the intrinsic molecular picture, conceptual DFT descriptors complement it by revealing how solvation modulates frontier molecular orbital energies as well as global and local reactivity.

Importantly, the incorporation of the Topological Analysis of the Fukui Function (TAFF) unified both perspectives by mapping nucleophilic, electrophilic, and radical susceptibilities directly onto QTAIM basins. The TAFF results confirmed that cleavage prone bonds identified as weak by QTAIM are also the most reactive according to the to *f*^+^, *f*^−^, and *f*^0^ indices, since the topological distribution of the Fukui functions reflects the underlying electron density. This overlap validates their mechanistic role in pesticide degradation.

Together, this integrated framework QTAIM for intrinsic bond lability, conceptual DFT for solvent dependent changes in frontier orbital energies and reactivity descriptors, and TAFF for topologically resolved reactivity provides a robust mechanistic understanding of HHP degradation and offers valuable insights for risk assessment, remediation strategies, and the rational design of safer pesticides.

## Data Availability

The original data presented in the study are openly available in University of Hertfordshire’s Pesticide Properties DataBase (PPDB) [39] and WHO hazard classification criteria [16].

## Supplementary Materials

The following supporting information can be downloaded at: https://www.mdpi.com/article/10.3390/toxics13100839/s1, Figure S1: Optimized structure of Diazinon in the gas phase highlighting the bond critical points (BCPs, type (3,−1), orange spheres) obtained from QTAIM topological analysis of the electron density; Table S1: Critical points (CP) and selected QTAIM descriptors, including Laplacian Bond Order (LBO), electron density at the bond critical point (ρ BCP) in a.u., and Laplacian of the electron density (∇^2^ρ BCP) in a.u., calculated for the bonds of Diazinon in the gas phase; Figure S2: Optimized structure of Diazinon in water as dielectric continuum highlighting the bond critical points (BCPs, type (3,−1), orange spheres) obtained from QTAIM topological analysis of the electron density; Table S2: Critical points (CP) and selected QTAIM descriptors, including Laplacian Bond Order (LBO), electron density at the bond critical point (ρ BCP) in a.u., and Laplacian of the electron density (∇^2^ρ BCP) in a.u., calculated for the bonds of Diazinon with PCM using water as continuum; Figure S3: Optimized structure of Diazinon supermolecule (surrounded by four water molecules) in water as dielectric continuum highlighting the bond critical points (BCPs, type (3,−1), orange spheres) obtained from QTAIM topological analysis of the electron density; Table S3: Critical points (CP) and selected QTAIM descriptors, including Laplacian Bond Order (LBO), electron density at the bond critical point (ρ BCP) in a.u., and Laplacian of the electron density (∇^2^ρ BCP) in a.u., calculated for the bonds of Diazinon supermolecule in PCM using water as continuum; Figure S4: Optimized structure of Chlorpyrifos in the gas phase highlighting the bond critical points (BCPs, type (3,−1), orange spheres) obtained from QTAIM topological analysis of the electron density; Table S4: Critical points (CP) and selected QTAIM descriptors, including Laplacian Bond Order (LBO), electron density at the bond critical point (ρ BCP) in a.u., and Laplacian of the electron density (∇^2^ρ BCP) in a.u., calculated for the bonds of Chlorpyrifos in the gas phase; Figure S5: Optimized structure of Chlorpyrifos in water as dielectric continuum highlighting the bond critical points (BCPs, type (3,−1), orange spheres) obtained from QTAIM topological analysis of the electron density; Table S5: Critical points (CP) and selected QTAIM descriptors, including Laplacian Bond Order (LBO), electron density at the bond critical point (ρ BCP) in a.u., and Laplacian of the electron density (∇^2^ρ BCP) in a.u., calculated for the bonds of Chlorpyrifos with PCM using water as continuum; Figure S6: Optimized structure of Chlorpyrifos supermolecule (surrounded by four water molecules) in water as dielectric continuum highlighting the bond critical points (BCPs, type (3,−1), orange spheres) obtained from QTAIM topological analysis of the electron density; Table S6: Critical points (CP) and selected QTAIM descriptors, including Laplacian Bond Order (LBO), electron density at the bond critical point (ρ BCP) in a.u., and Laplacian of the electron density (∇^2^ρ BCP) in a.u., calculated for the bonds of Chlorpyrifos supermolecule in PCM using water as continuum; Figure S7: Optimized structure of Acephate in the gas phase highlighting the bond critical points (BCPs, type (3,−1), orange spheres) obtained from QTAIM topological analysis of the electron density; Table S7: Critical points (CP) and selected QTAIM descriptors, including Laplacian Bond Order (LBO), electron density at the bond critical point (ρ BCP) in a.u., and Laplacian of the electron density (∇^2^ρ BCP) in a.u., calculated for the bonds of Acephate in the gas phase; Figure S8: Optimized structure of Acephate in water as dielectric continuum highlighting the bond critical points (BCPs, type (3,−1), orange spheres) obtained from QTAIM topological analysis of the electron density; Table S8: Critical points (CP) and selected QTAIM descriptors, including Laplacian Bond Order (LBO), electron density at the bond critical point (ρ BCP) in a.u., and Laplacian of the electron density (∇^2^ρ BCP) in a.u., calculated for the bonds of Acephate supermolecule in PCM using water as continuum; Figure S9: Optimized structure of Acephate supermolecule (surrounded by four water molecules) in water as dielectric continuum highlighting the bond critical points (BCPs, type (3,–1), orange spheres) obtained from QTAIM topological analysis of the electron density; Table S9: Critical points (CP) and selected QTAIM descriptors, including Laplacian Bond Order (LBO), electron density at the bond critical point (ρ BCP) in a.u., and Laplacian of the electron density (∇^2^ρ BCP) in a.u., calculated for the bonds of Acephate supermolecule in PCM using water as continuum; Figure S10: Optimized structure of Methamidophos in the gas phase highlighting the bond critical points (BCPs, type (3,−1), orange spheres) obtained from QTAIM topological analysis of the electron density; Table S10: Critical points (CP) and selected QTAIM descriptors, including Laplacian Bond Order (LBO), electron density at the bond critical point (ρ BCP) in a.u., and Laplacian of the electron density (∇^2^ρ BCP) in a.u., calculated for the bonds of Methamidophos in the gas phase; Figure S11: Optimized structure of Methamidophos in water as dielectric continuum highlighting the bond critical points (BCPs, type (3,−1), orange spheres) obtained from QTAIM topological analysis of the electron density; Table S11: Critical points (CP) and selected QTAIM descriptors, including Laplacian Bond Order (LBO), electron density at the bond critical point (ρ BCP) in a.u., and Laplacian of the electron density (∇^2^ρ BCP) in a.u., calculated for the bonds of Methamidophos supermolecule in PCM using water as continuum; Figure S12: Optimized structure of Methamidophos supermolecule (surrounded by four water molecules) in water as dielectric continuum highlighting the bond critical points (BCPs, type (3,−1), orange spheres) obtained from QTAIM topological analysis of the electron density; Table S12: Critical points (CP) and selected QTAIM descriptors, including Laplacian Bond Order (LBO), electron density at the bond critical point (ρ BCP) in a.u., and Laplacian of the electron density (∇^2^ρ BCP) in a.u., calculated for the bonds of Methamidophos supermolecule in PCM using water as continuum; Figure S13: Non-Covalent Interaction (NCI) analysis of the diazinon–water supermolecule. The isosurfaces are color-mapped according to the sign(λ_2_)ρ scale: strong attractive interactions (hydrogen bonding) appear in blue. weaker van der Waals interactions in green. and steric or repulsive interactions in red. The corresponding scatter plot (bottom) shows the relationship between the reduced density gradient (RDG) and sign(λ_2_)ρ. allowing a quantitative distinction of interaction types; Figure S14: Non-Covalent Interaction (NCI) analysis of the chlorpyrifos–water supermolecule. The isosurfaces are color-mapped according to the sign(λ_2_)ρ scale: strong attractive interactions (hydrogen bonding) appear in blue. weaker van der Waals interactions in green. and steric or repulsive interactions in red. The corresponding scatter plot (bottom) shows the relationship between the reduced density gradient (RDG) and sign(λ_2_)ρ. allowing a quantitative distinction of interaction types; Figure S15: Non-Covalent Interaction (NCI) analysis of the acephate–water supermolecule. The isosurfaces are color-mapped according to the sign(λ_2_)ρ scale: strong attractive interactions (hydrogen bonding) appear in blue. weaker van der Waals interactions in green. and steric or repulsive interactions in red. The corresponding scatter plot (bottom) shows the relationship between the reduced density gradient (RDG) and sign(λ_2_)ρ. allowing a quantitative distinction of interaction types; Figure S16: Non-Covalent Interaction (NCI) analysis of the methamidophos–water supermolecule. The isosurfaces are color-mapped according to the sign(λ_2_)ρ scale: strong attractive interactions (hydrogen bonding) appear in blue. weaker van der Waals interactions in green. and steric or repulsive interactions in red. The corresponding scatter plot (bottom) shows the relationship between the reduced density gradient (RDG) and sign(λ_2_)ρ. allowing a quantitative distinction of interaction types; Table S13: HOMO, LUMO, η, μ, and ω values (eV) for diazinon, chlorpyrifos, acephate, and methamidophos in gas phase, PCM water, and supermolecule in PCM; Table S14: Dipole moments (Debye) and total energies (kcal/mol) of diazinon, chlorpyrifos, acephate, and methamidophos in gas phase, PCM water, and supermolecule in PCM; Table S15: Condensed electrophilic Fukui functions (*f*^+^) for diazinon in gas phase, PCM water, and supermolecule in PCM; Table S16: Condensed electrophilic Fukui functions (*f*^+^) for chlorpyrifos in gas phase, PCM water, and supermolecule in PCM; Table S17: Condensed electrophilic Fukui functions (*f*^+^) for acephate in gas phase, PCM water, and supermolecule in PCM; Table S18: Condensed electrophilic Fukui functions (*f*^+^) for methamidophos in gas phase, PCM water, and supermolecule in PCM.
