# Supplementary material for: QTAIM Based Computational Assessment of Cleavage Prone Bonds in Highly Hazardous Pesticides
Source: Toxics. 2025 Oct 1;13(10):839. doi: 10.3390/toxics13100839 (PMC12568069; doi:10.3390/toxics13100839)
Supplement: Supplementary file 1 [file toxics-13-00839-s001.zip › toxics-3864358-supplementary.pdf]

Electronic Supplementary Material (ESI)

Supporting Information

QTAIM Based Computational Assessment of Cleavage Prone Bonds in Highly Hazardous Pesticides

Andrés Aracena*. Sebastián Elgueta. Sebastián Pizarro and César Zúñiga

Table S1. Critical points (CP) and selected QTAIM descriptors. including Laplacian Bond Order (LBO). electron density at the bond critical point (ρ BCP) in a.u.. and Laplacian of the electron density ($\nabla^2\rho$ BCP) in a.u.. calculated for the bonds of Diazinon in the gas phase.

CP	Selected bond	(LBO)	(ρ BCP)	($\nabla^2\rho$ BCP)
**27**	6(N) 12(C)	1.293	0.363	-1.150
**26**	7(N) 9(C)	1.204	0.346	-1.040
**37**	7(N) 13(C)	1.149	0.338	-1.020
**2**	16(C) 31(H)	0.836	0.286	-0.995
**25**	17(C) 33(H)	0.836	0.286	-0.991
**5**	16(C) 32(H)	0.838	0.285	-0.984
**33**	17(C) 34(H)	0.834	0.284	-0.980
**39**	14(C) 27(H)	0.822	0.281	-0.965
**23**	6(N) 9(C)	1.126	0.335	-0.964
**18**	8(C) 20(H)	0.800	0.280	-0.947
**41**	15(C) 29(H)	0.812	0.278	-0.938
**3**	18(C) 37(H)	0.807	0.275	-0.919
**7**	18(C) 36(H)	0.808	0.275	-0.919
**11**	11(C) 26(H)	0.804	0.276	-0.918
**36**	19(C) 39(H)	0.808	0.275	-0.918
**29**	19(C) 40(H)	0.807	0.275	-0.917
**10**	10(C) 21(H)	0.802	0.275	-0.914
**16**	11(C) 24(H)	0.803	0.274	-0.908
**14**	10(C) 23(H)	0.804	0.273	-0.906
**35**	19(C) 38(H)	0.806	0.273	-0.904
**1**	18(C) 35(H)	0.800	0.273	-0.904
**6**	10(C) 22(H)	0.801	0.273	-0.903
**8**	11(C) 25(H)	0.799	0.273	-0.902
**43**	15(C) 30(H)	0.795	0.272	-0.897
**42**	15(C) 28(H)	0.793	0.272	-0.897

**34**	12(C) 14(C)	1.453	0.314	-0.894
**38**	13(C) 14(C)	1.453	0.313	-0.874
**40**	13(C) 15(C)	1.055	0.256	-0.624
**21**	8(C) 9(C)	1.015	0.256	-0.623
**4**	16(C) 18(C)	1.043	0.252	-0.609
**31**	17(C) 19(C)	1.043	0.252	-0.607
**15**	8(C) 10(C)	0.898	0.236	-0.520
**17**	8(C) 11(C)	0.889	0.235	-0.515
**30**	3(O) 12(C)	0.376	0.275	-0.317
**19**	1(S) 2(P)	1.121	0.177	-0.306
**28**	5(O) 17(C)	0.192	0.224	-0.277
**13**	4(O) 16(C)	0.184	0.221	-0.256
**24**	2(P) 3(O)	0.310	0.162	0.535
**22**	2(P) 5(O)	0.413	0.180	0.700
**20**	2(P) 4(O)	0.427	0.183	0.747

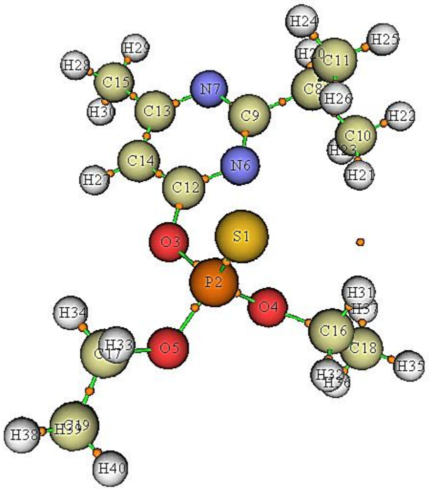

Figure S1: Optimized structure of Diazinon in the gas phase highlighting the bond critical points (BCPs. type (3.–1). orange spheres) obtained from QTAIM topological analysis of the electron density.

Table S2. Critical points (CP) and selected QTAIM descriptors. including Laplacian Bond Order (LBO). electron density at the bond critical point (ρ BCP) in a.u.. and Laplacian of the electron density ($\nabla^2\rho$ BCP) in a.u.. calculated for the bonds of Diazinon with PCM using water as continuum.

**CP**	**Selected bond**	**(LBO)**	**(ρ BCP)**	**($\nabla^2\rho$ BCP)**
**56**	3(O) 12(C)	0.358	0.271	-0.301
**55**	5(O) 17(C)	0.168	0.216	-0.239
**73**	4(O) 16(C)	0.161	0.214	-0.234
**63**	1(S) 2(P)	1.081	0.174	-0.298
**60**	2(P) 3(O)	0.322	0.164	0.567
**62**	2(P) 5(O)	0.427	0.183	0.737

**67**	2(P) 4(O)	0.440	0.185	0.762

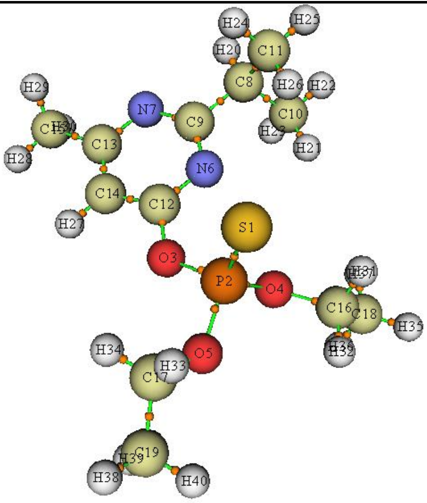

Figure S2: Optimized structure of Diazinon in water as dielectric continuum highlighting the bond critical points (BCPs. type (3.–1). orange spheres) obtained from QTAIM topological analysis of the electron density.

Table S3. Critical points (CP) and selected QTAIM descriptors. including Laplacian Bond Order (LBO). electron density at the bond critical point (ρ BCP) in a.u.. and Laplacian of the electron density ($\nabla^2\rho$ BCP) in a.u.. calculated for the bonds of Diazinon supermolecule in PCM using water as continuum.

CP	Selected bond	(LBO)	(ρ BCP)	($\nabla^2\rho$ BCP)
**86**	3(O) 12(C)	0.349	0.269	-0.338
**76**	5(O) 17(C)	0.149	0.210	-0.214
**105**	4(O) 16(C)	0.138	0.205	-0.226
**81**	1(S) 2(P)	1.102	0.177	-0.310
**87**	2(P) 3(O)	0.294	0.162	0.557
**84**	2(P) 5(O)	0.410	0.181	0.718
**91**	2(P) 4(O)	0.425	0.185	0.751

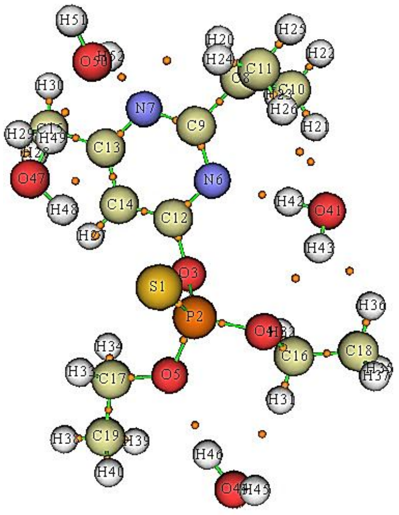

Figure S3: Optimized structure of Diazinon supermolecule (surrounded by four water molecules) in water as dielectric continuum highlighting the bond critical points (BCPs. type (3.–1). orange spheres) obtained from QTAIM topological analysis of the electron density.

Table S4. Critical points (CP) and selected QTAIM descriptors. including Laplacian Bond Order (LBO). electron density at the bond critical point (ρ BCP) in a.u.. and Laplacian of the electron density ($\nabla^2\rho$ BCP) in a.u.. calculated for the bonds of Chlorpyrifos in the gas phase.

CP	Selected bond	(LBO)	(ρ BCP)	($\nabla^2\rho$ BCP)
**17**	9(N) 12(C)	1.216	0.352	-1.030
**6**	10(C) 19(H)	0.846	0.289	-1.010
**32**	16(C) 29(H)	0.840	0.285	-1.000
**3**	10(C) 20(H)	0.840	0.286	-0.995
**14**	9(N) 18(C)	1.206	0.350	-0.991
**19**	11(C) 21(H)	0.837	0.286	-0.990
**26**	11(C) 22(H)	0.838	0.285	-0.988
**27**	14(C) 27(H)	0.810	0.275	-0.920
**20**	14(C) 28(H)	0.809	0.275	-0.920
**5**	13(C) 25(H)	0.808	0.275	-0.919
**2**	13(C) 24(H)	0.810	0.275	-0.918
**29**	14(C) 26(H)	0.802	0.273	-0.904
**1**	13(C) 23(H)	0.803	0.273	-0.904
**30**	15(C) 16(C)	1.429	0.314	-0.880
**25**	12(C) 15(C)	1.369	0.308	-0.852
**28**	16(C) 17(C)	1.375	0.308	-0.851
**16**	17(C) 18(C)	1.351	0.308	-0.836
**4**	10(C) 13(C)	1.049	0.253	-0.611
**24**	11(C) 14(C)	1.041	0.252	-0.609
**21**	8(O) 12(C)	0.439	0.292	-0.353
**10**	4(S) 5(P)	1.127	0.178	-0.307

**23**	2(Cl) 17(C)	0.584	0.200	-0.290
**31**	1(Cl) 15(C)	0.588	0.200	-0.289
**12**	3(Cl) 18(C)	0.598	0.200	-0.287
**18**	7(O) 11(C)	0.182	0.220	-0.255
**7**	6(O) 10(C)	0.166	0.215	-0.215
**15**	5(P) 8(O)	0.257	0.152	0.442
**13**	5(P) 7(O)	0.441	0.184	0.748
**11**	5(P) 6(O)	0.440	0.185	0.774

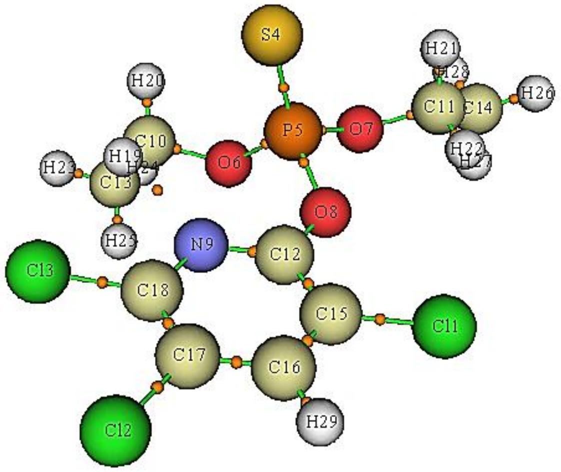

Figure S4: Optimized structure of Chlorpyrifos in the gas phase highlighting the bond critical points (BCPs. type (3.–1). orange spheres) obtained from QTAIM topological analysis of the electron density.

Table S5. Critical points (CP) and selected QTAIM descriptors. including Laplacian Bond Order (LBO). electron density at the bond critical point (ρ BCP) in a.u.. and Laplacian of the electron density ($\nabla^2\rho$ BCP) in a.u.. calculated for the bonds of Chlorpyrifos with PCM using water as continuum.

CP	Selected bond	(LBO)	(ρ BCP)	($\nabla^2\rho$ BCP)
**42**	8(O) 12(C)	0.416	0.286	-0.335
**38**	2(Cl) 17(C)	0.585	0.200	-0.291
**31**	1(Cl) 15(C)	0.591	0.200	-0.291
**51**	3(Cl) 18(C)	0.590	0.199	-0.284
**43**	7(O) 11(C)	0.160	0.213	-0.225
**55**	6(O) 10(C)	0.151	0.209	-0.193
**50**	4(S) 5(P)	1.096	0.175	-0.299
**47**	5(P) 8(O)	0.274	0.156	0.480
**49**	5(P) 7(O)	0.450	0.186	0.767
**52**	5(P) 6(O)	0.442	0.186	0.786

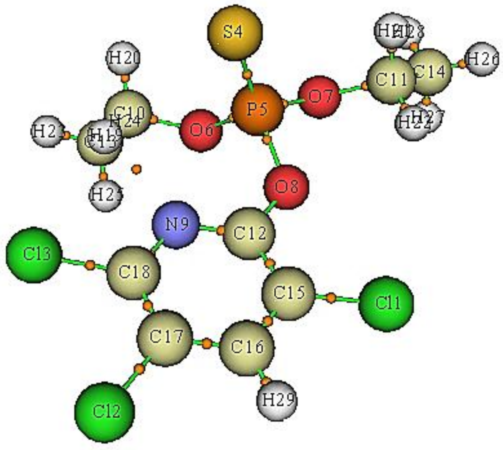

Figure S5: Optimized structure of Chlorpyrifos in water as dielectric continuum highlighting the bond critical points (BCPs. type (3.–1). orange spheres) obtained from QTAIM topological analysis of the electron density.

Table S6. Critical points (CP) and selected QTAIM descriptors. including Laplacian Bond Order (LBO). electron density at the bond critical point (ρ BCP) in a.u.. and Laplacian of the electron density ($\nabla^2\rho$ BCP) in a.u.. calculated for the bonds of Chlorpyrifos supermolecule in PCM using water as continuum.

CP	Selected bond	(LBO)	(ρ BCP)	($\nabla^2\rho$ BCP)
**64**	8(O) 12(C)	0.400	0.282	-0.304
**60**	2(Cl) 17(C)	0.586	0.200	-0.292
**53**	1(Cl) 15(C)	0.591	0.201	-0.290
**75**	3(Cl) 18(C)	0.584	0.199	-0.286
**77**	7(O) 11(C)	0.148	0.210	-0.203
**84**	6(O) 10(C)	0.149	0.210	-0.198
**81**	4(S) 5(P)	1.096	0.176	-0.301
**73**	5(P) 8(O)	0.291	0.160	0.519
**76**	5(P) 7(O)	0.412	0.181	0.710
**79**	5(P) 6(O)	0.423	0.184	0.765

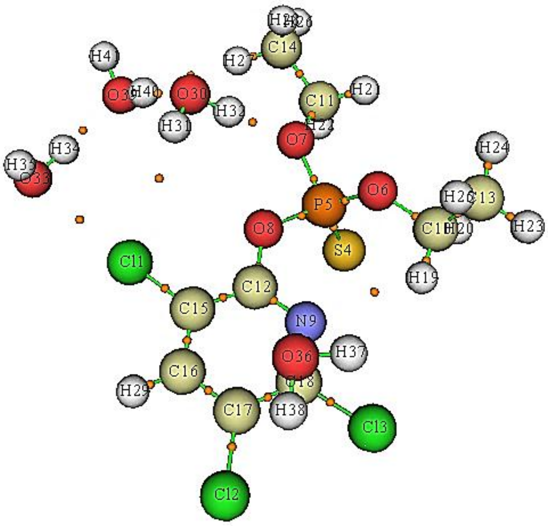

Figure S6: Optimized structure of Chlorpyrifos supermolecule (surrounded by four water molecules) in water as dielectric continuum highlighting the bond critical points (BCPs. type (3.–1). orange spheres) obtained from QTAIM topological analysis of the electron density.

Table S7. Critical points (CP) and selected QTAIM descriptors. including Laplacian Bond Order (LBO). electron density at the bond critical point (ρ BCP) in a.u.. and Laplacian of the electron density ($\nabla^2\rho$ BCP) in a.u.. calculated for the bonds of Acephate in the gas phase.

CP	Selected bond	(LBO)	(ρ BCP)	($\nabla^2\rho$ BCP)
**20**	10(C) 19(H)	0.847	0.286	-0.996
**21**	10(C) 20(H)	0.853	0.285	-0.995
**19**	10(C) 18(H)	0.839	0.282	-0.967
**2**	9(C) 17(H)	0.826	0.281	-0.961
**10**	9(C) 16(H)	0.831	0.280	-0.950
**7**	8(C) 13(H)	0.814	0.279	-0.943
**3**	9(C) 15(H)	0.822	0.278	-0.938
**5**	8(C) 14(H)	0.797	0.273	-0.900
**1**	8(C) 12(H)	0.788	0.271	-0.894
**13**	6(N) 7(C)	0.804	0.291	-0.809
**8**	7(C) 8(C)	1.022	0.252	-0.603
**18**	3(O) 10(C)	0.213	0.228	-0.237
**6**	1(S) 9(C)	0.540	0.168	-0.229
**11**	1(S) 2(P)	0.695	0.135	-0.198
**14**	5(O) 7(C)	1.131	0.416	-0.196
**4**	6(N) 11(H)	0.663	0.336	-0.158
**12**	2(P) 6(N)	0.653	0.165	0.271
**15**	2(P) 3(O)	0.428	0.181	0.769
**9**	2(P) 4(O)	0.739	0.235	1.490

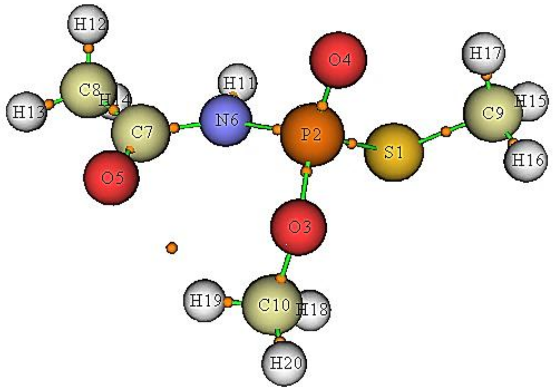

Figure S7: Optimized structure of Acephate in the gas phase highlighting the bond critical points (BCPs. type (3.–1). orange spheres) obtained from QTAIM topological analysis of the electron density.

Table S8. Critical points (CP) and selected QTAIM descriptors. including Laplacian Bond Order (LBO). electron density at the bond critical point (ρ BCP) in a.u.. and Laplacian of the electron density (∇²ρ BCP) in a.u.. calculated for the bonds of Acephate supermolecule in PCM using water as continuum.

CP	Selected bond	(LBO)	(ρ BCP)	(∇²ρ BCP)
**36**	3(O) 10(C)	0.206	0.225	-0.238
**25**	1(S) 9(C)	0.530	0.167	-0.227
**31**	1(S) 2(P)	0.725	0.138	-0.207
**33**	6(N) 7(C)	0.834	0.295	-0.833
**32**	2(P) 6(N)	0.698	0.170	0.301
**35**	2(P) 3(O)	0.414	0.179	0.726
**28**	2(P) 4(O)	0.714	0.231	1.425

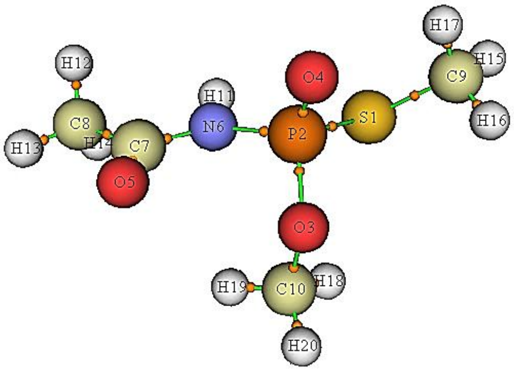

Figure S8: Optimized structure of Acephate in water as dielectric continuum highlighting the bond critical points (BCPs. type (3.–1). orange spheres) obtained from QTAIM topological analysis of the electron density.

Table S9. Critical points (CP) and selected QTAIM descriptors. including Laplacian Bond Order (LBO). electron density at the bond critical point (ρ BCP) in a.u.. and Laplacian of the electron density ($\nabla^2\rho$ BCP) in a.u.. calculated for the bonds of Acephate supermolecule in PCM using water as continuum.

CP	Selected bond	(LBO)	(ρ BCP)	($\nabla^2\rho$ BCP)
**51**	3(O) 10(C)	0.182	0.218	-0.220
**46**	1(S) 9(C)	0.517	0.167	-0.224
**47**	1(S) 2(P)	0.759	0.148	-0.213
**44**	6(N) 7(C)	0.884	0.303	-0.869
**53**	2(P) 6(N)	0.747	0.176	0.331
**52**	2(P) 3(O)	0.432	0.181	0.741
**59**	2(P) 4(O)	0.648	0.223	1.306

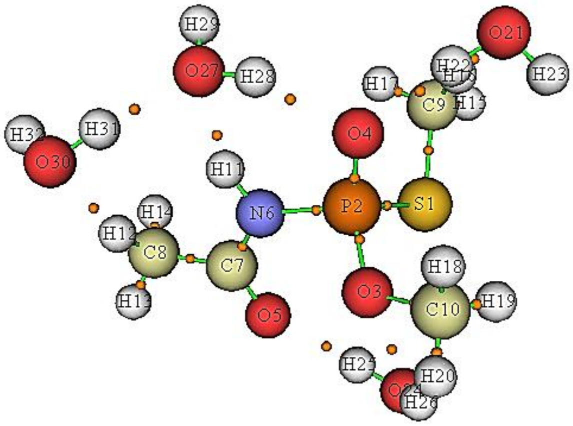

Figure S9: Optimized structure of Acephate supermolecule (surrounded by four water molecules) in water as dielectric continuum highlighting the bond critical points (BCPs. type (3.–1). orange spheres) obtained from QTAIM topological analysis of the electron density.

Table S10. Critical points (CP) and selected QTAIM descriptors. including Laplacian Bond Order (LBO). electron density at the bond critical point (ρ BCP) in a.u.. and Laplacian of the electron density ($\nabla^2\rho$ BCP) in a.u.. calculated for the bonds of Methamidophos in the gas phase.

CP	Selected bond	(LBO)	(ρ BCP)	($\nabla^2\rho$ BCP)
**2**	5(N) 8(H)	0.662	0.334	-1.540
**3**	5(N) 9(H)	0.657	0.332	-1.500
**13**	7(C) 14(H)	0.851	0.285	-0.991
**14**	7(C) 15(H)	0.840	0.283	-0.973
**12**	7(C) 13(H)	0.839	0.283	-0.970
**4**	6(C) 12(H)	0.828	0.281	-0.961
**9**	6(C) 11(H)	0.829	0.280	-0.947
**8**	6(C) 10(H)	0.823	0.278	-0.937

**11**	3(O) 7(C)	0.231	0.233	-0.296
**10**	1(S) 6(C)	0.550	0.168	-0.230
**7**	1(S) 2(P)	0.719	0.136	-0.204
**5**	2(P) 5(N)	0.832	0.178	0.362
**6**	2(P) 3(O)	0.389	0.174	0.674
**1**	2(P) 4(O)	0.737	0.233	1.470

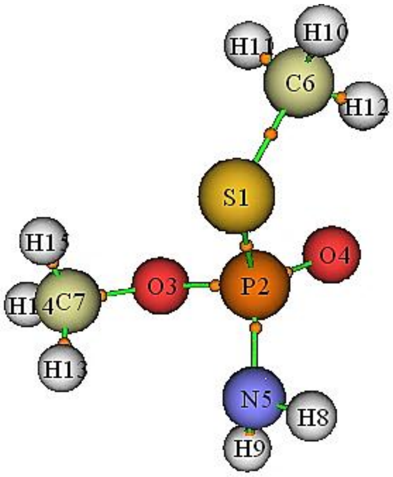

Figure S10: Optimized structure of Methamidophos in the gas phase highlighting the bond critical points (BCPs. type (3.–1). orange spheres) obtained from QTAIM topological analysis of the electron density.

Table S11. Critical points (CP) and selected QTAIM descriptors. including Laplacian Bond Order (LBO). electron density at the bond critical point (ρ BCP) in a.u.. and Laplacian of the electron density ($\nabla^2\rho$ BCP) in a.u.. calculated for the bonds of Methamidophos supermolecule in PCM using water as continuum.

CP	Selected bond	(LBO)	(ρ BCP)	($\nabla^2\rho$ BCP)
**25**	3(O) 7(C)	0.213	0.227	-0.262
**23**	1(S) 6(C)	0.538	0.168	-0.232
**21**	1(S) 2(P)	0.732	0.138	-0.211
**53**	2(P) 5(N)	0.877	0.183	0.402
**18**	2(P) 3(O)	0.394	0.175	0.684
**19**	2(P) 4(O)	0.704	0.229	1.399

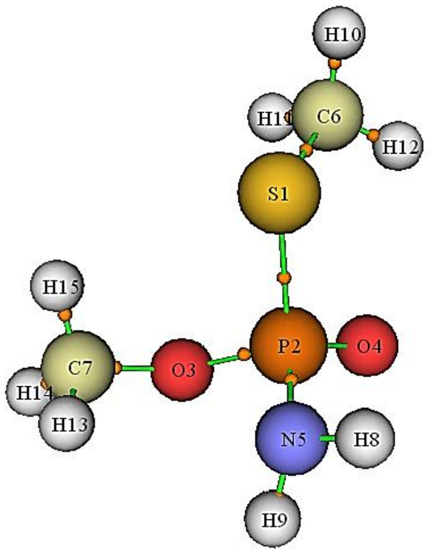

Figure S11: Optimized structure of Methamidophos in water as dielectric continuum highlighting the bond critical points (BCPs. type (3.–1). orange spheres) obtained from QTAIM topological analysis of the electron density.

Table S12. Critical points (CP) and selected QTAIM descriptors. including Laplacian Bond Order (LBO). electron density at the bond critical point (ρ BCP) in a.u.. and Laplacian of the electron density ($\nabla^2\rho$ BCP) in a.u.. calculated for the bonds of Methamidophos supermolecule in PCM using water as continuum.

CP	Selected bond	(LBO)	(ρ BCP)	($\nabla^2\rho$ BCP)
**40**	3(O) 7(C)	0.202	0.224	-0.241
**30**	1(S) 6(C)	0.521	0.167	-0.226
**36**	1(S) 2(P)	0.730	0.138	-0.213
**46**	2(P) 5(N)	0.923	0.191	0.445
**44**	2(P) 3(O)	0.402	0.176	0.699
**50**	2(P) 4(O)	0.649	0.221	1.276

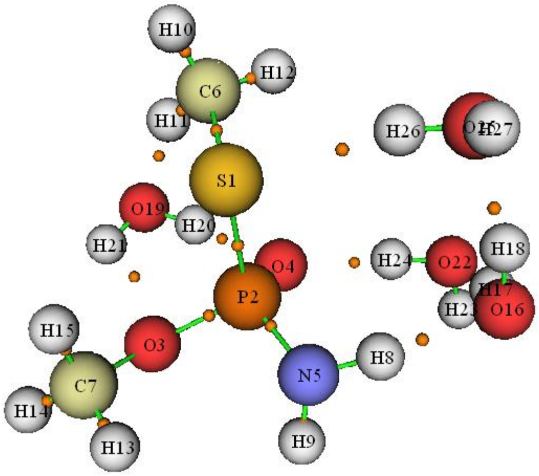

Figure S12: Optimized structure of Methamidophos supermolecule (surrounded by four water molecules) in water as dielectric continuum highlighting the bond critical points (BCPs. type (3.–1). orange spheres) obtained from QTAIM topological analysis of the electron density.

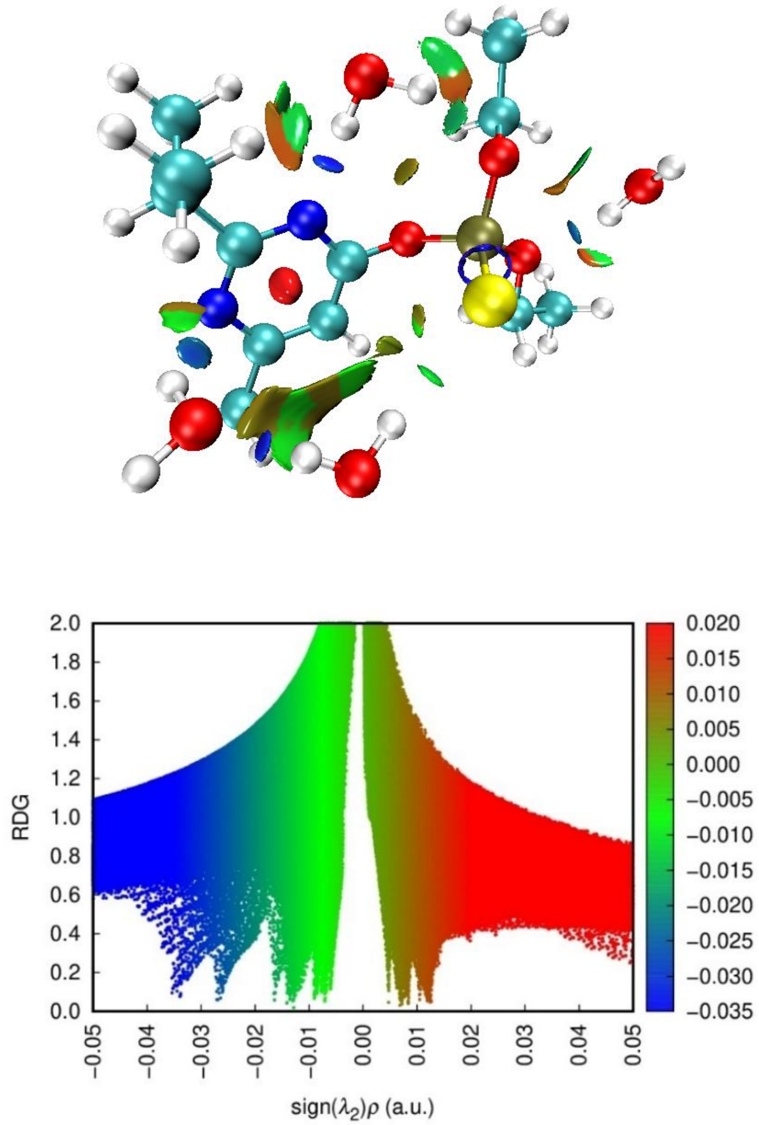

Figure S13: Non-Covalent Interaction (NCI) analysis of the diazinon–water supermolecule. The isosurfaces are color-mapped according to the sign(λ_2)ρ scale: strong attractive interactions (hydrogen bonding) appear in blue. weaker van der Waals interactions in green. and steric or repulsive interactions in red. The corresponding scatter plot (bottom) shows the relationship between the reduced density gradient (RDG) and sign(λ_2)ρ. allowing a quantitative distinction of interaction types.

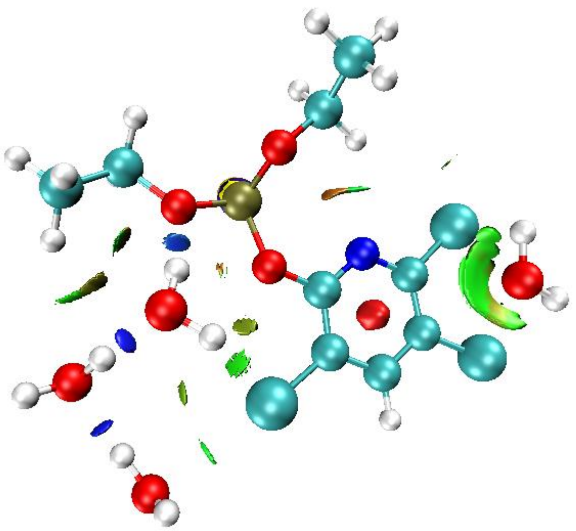

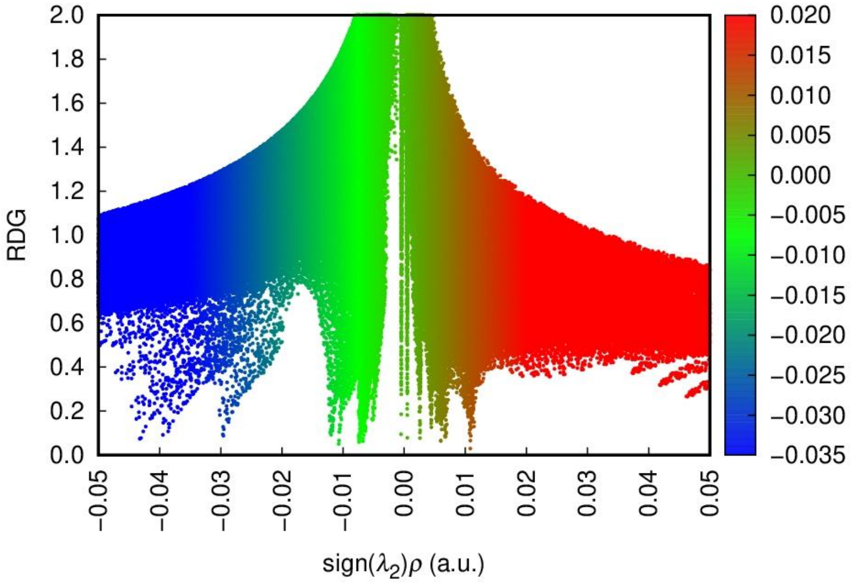

Figure S14: Non-Covalent Interaction (NCI) analysis of the chlorpyrifos–water supermolecule. The isosurfaces are color-mapped according to the sign(λ_2)ρ scale: strong attractive interactions (hydrogen bonding) appear in blue. weaker van der Waals interactions in green. and steric or repulsive interactions in red. The corresponding scatter plot (bottom) shows the relationship between the reduced density gradient (RDG) and sign(λ_2)ρ. allowing a quantitative distinction of interaction types.

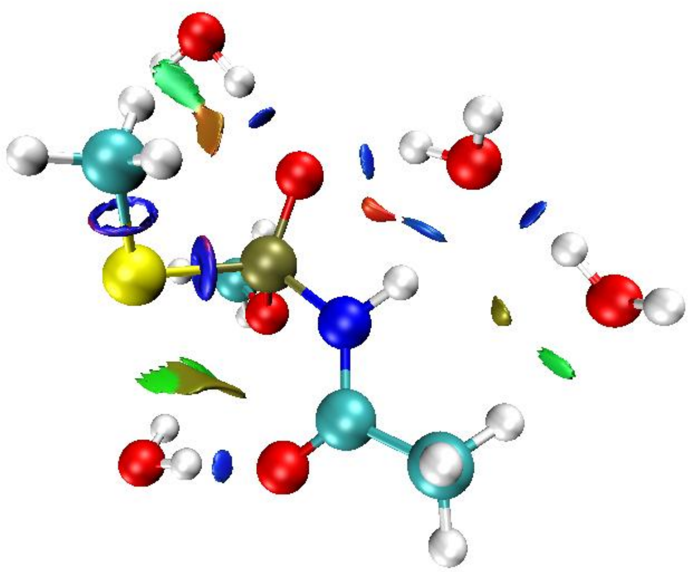

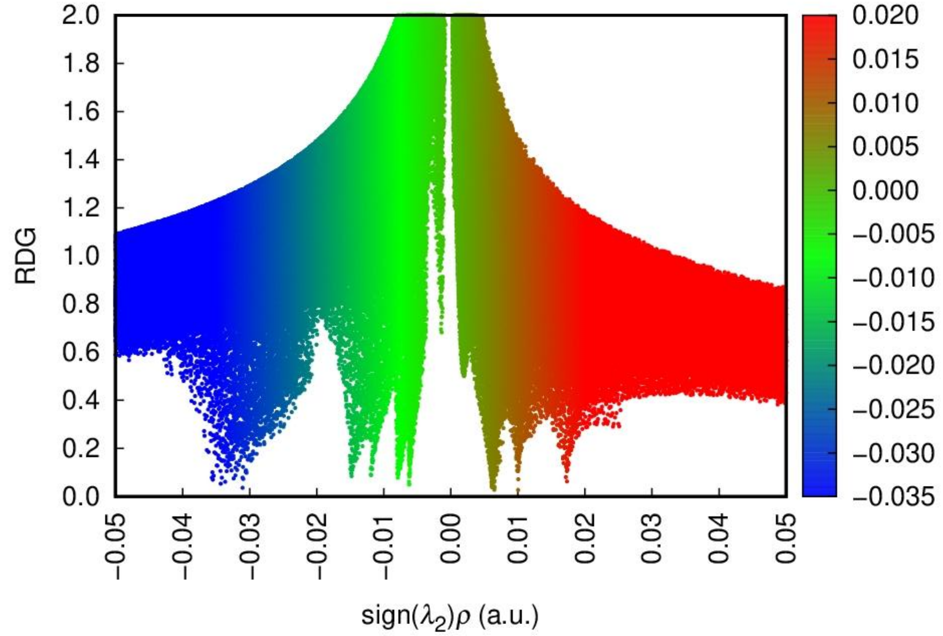

Figure S15: Non-Covalent Interaction (NCI) analysis of the acephate–water supermolecule. The isosurfaces are color-mapped according to the sign(λ_2)ρ scale: strong attractive interactions (hydrogen bonding) appear in blue. weaker van der Waals interactions in green. and steric or repulsive interactions in red. The corresponding scatter plot (bottom) shows the relationship between the reduced density gradient (RDG) and sign(λ_2)ρ. allowing a quantitative distinction of interaction types.

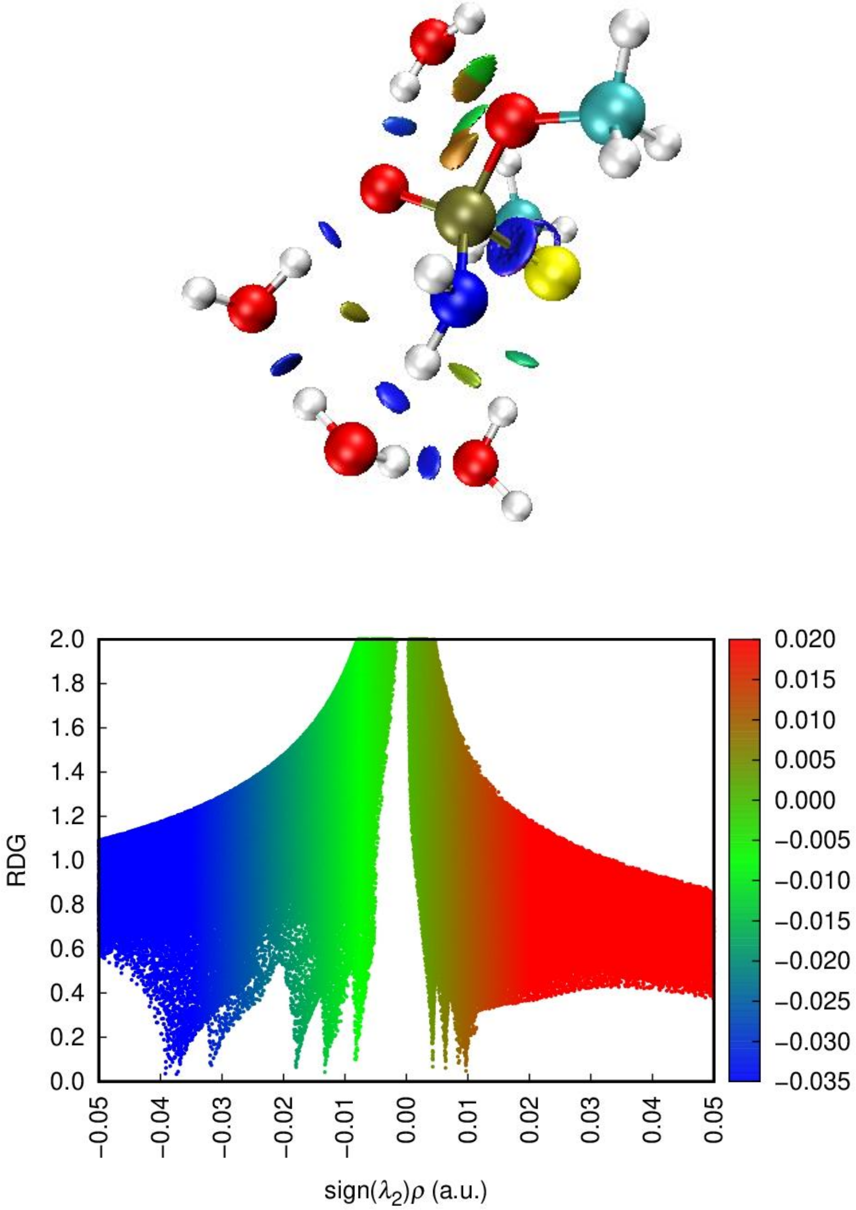

Figure S16: Non-Covalent Interaction (NCI) analysis of the methamidophos–water supermolecule. The isosurfaces are color-mapped according to the sign(λ_2)ρ scale: strong attractive interactions (hydrogen bonding) appear in blue. weaker van der Waals interactions in green. and steric or repulsive interactions in red. The corresponding scatter plot (bottom) shows the relationship between the reduced density gradient (RDG) and sign(λ_2)ρ. allowing a quantitative distinction of interaction types.

Table S13. HOMO, LUMO, η, μ, and ω values (eV) for diazinon, chlorpyrifos, acephate, and methamidophos in gas phase, PCM water, and supermolecule in PCM.

	HOMO (eV)	LUMO (eV)	η (eV)	μ (eV)	ω (eV)
**Diazinon**					
Gas phase	-6.607	-1.060	5.547	-3.833	1.324
PCM water	-7.028	-1.224	5.804	-4.126	1.467
Supermolecule in PCM	-6.935	-1.255	5.680	-4.095	1.476
**Chlorpyrifos**					
Gas phase	-6.875	-1.954	4.921	-4.414	1.980
PCM water	-7.065	-1.883	5.182	-4.474	1.931
Supermolecule in PCM	-7.087	-1.860	5.227	-4.474	1.915
**Acephate**					
Gas phase	-7.201	-0.632	6.569	-3.917	1.168
PCM water	-7.283	-0.613	6.670	-3.948	1.168
Supermolecule in PCM	-7.307	-0.702	6.606	-4.005	1.214
**Methamidophos**					
Gas phase	-7.045	-0.002	7.043	-3.524	0.881
PCM water	-7.145	-0.032	7.112	-3.589	0.905
Supermolecule in PCM	-7.386	-0.238	7.148	-3.812	1.017

Table S14. Dipole moments (Debye) and total energies (kcal/mol) of diazinon, chlorpyrifos, acephate, and methamidophos in gas phase, PCM water, and supermolecule in PCM.

Diazinon	Dipole moment (Debye)	Total energy (kcal/mol)
Gas phase	2.667	-969483.0
PCM water	4.316	-969490.7
Supermolecule in PCM	7.888	-1161413.4
**Chlorpyrifos**		
Gas phase	2.280	-1725925.0
PCM water	3.567	-1725930.9
Supermolecule in PCM	7.931	-1917853.3
**Acephate**		
Gas phase	4.273	-739604.7
PCM water	7.509	-739616.3
Supermolecule in PCM	4.665	-931547.8
**Methamidophos**		
Gas phase	3.022	-643791.6
PCM water	4.255	-643800.8
Supermolecule in PCM	4.665	-931547.8

Table S15. Condensed electrophilic Fukui functions (f^{+}) for diazinon in gas phase, PCM water, and supermolecule in PCM.

Diazinon	Gas Phase	PCM water	Supermolecule in PCM
ATOM# 1S	0.0037	0.0083	0.0132
ATOM# 2P	0.0074	0.0165	0.0204
ATOM# 3O	0.0047	0.0151	0.0112
ATOM# 4O	0.0001	0.0003	0.0018
ATOM# 5O	0.0002	0.0009	0.0021
ATOM# 6N	0.2073	0.2086	0.1980
ATOM# 7N	0.0254	0.0838	0.1069
ATOM# 8C	0.0093	0.0049	0.0035
ATOM# 9C	0.1513	0.0407	0.0255
ATOM# 10C	0.0068	0.0031	0.0026
ATOM# 11C	0.0075	0.0030	0.0022
ATOM# 12C	0.0532	0.1645	0.2014
ATOM# 13C	0.3321	0.3452	0.3423
ATOM# 14C	0.1307	0.0487	0.0185
ATOM# 15C	0.0081	0.0078	0.0054
ATOM# 16C	0.0007	0.0010	0.0001
ATOM# 17C	0.0001	0.0002	0.0001
ATOM# 18C	0	0	0.0001
ATOM# 19C	0	0.0001	0
ATOM# 20H	0	0	0.0001
ATOM# 21H	0.0001	0.0003	0.0001
ATOM# 22H	0.0008	0	0.0001
ATOM# 23H	0.0012	0.0006	0.0002
ATOM# 24H	0.0012	0.0006	0.0005
ATOM# 25H	0.0014	0	0
ATOM# 26H	0.0001	0.0003	0.0002
ATOM# 27H	0.0008	0.0003	0.0001
ATOM# 28H	0.0230	0	0.0094
ATOM# 29H	0	0.0228	0.0230
ATOM# 30H	0.0227	0.0231	0.0029
ATOM# 31H	0	0.0001	0.0001
ATOM# 32H	0	0	0.0001
ATOM# 33H	0	0	0
ATOM# 34H	0	0.0001	0.0001
ATOM# 35H	0	0.0001	0
ATOM# 36H	0	0	0
ATOM# 37H	0	0	0
ATOM# 38H	0	0	0.0001
ATOM# 39H	0	0	0

ATOM# 40H	0	0	0

Table S16. Condensed electrophilic Fukui functions (f^{+}) for chlorpyrifos in gas phase, PCM water, and supermolecule in PCM.

Chlorpyrifos	Gas Phase	PCM water	Supermolecule in PCM
ATOM# 1Cl	0.0156	0.0157	0.0149
ATOM# 2Cl	0.0017	0.0018	0.0021
ATOM# 3Cl	0.0187	0.0186	0.0163
ATOM# 4S	0.0042	0.0038	0.0047
ATOM# 5P	0.0059	0.0051	0.0073
ATOM# 6O	0	0	0.0001
ATOM# 7O	0.0001	0.0002	0.0004
ATOM# 8O	0.0052	0.0050	0.0057
ATOM# 9N	0.2213	0.2216	0.2273
ATOM# 10C	0.0005	0.0006	0.0006
ATOM# 11C	0.0001	0.0001	0
ATOM# 12C	0.0491	0.0488	0.0621
ATOM# 13C	0	0.0001	0
ATOM# 14C	0	0	0
ATOM# 15C	0.1483	0.1485	0.1313
ATOM# 16C	0.3311	0.3323	0.3320
ATOM# 17C	0.0258	0.0260	0.0296
ATOM# 18C	0.1701	0.1698	0.1624
ATOM# 19H	0	0.0001	0.0001
ATOM# 20H	0	0	0
ATOM# 21H	0	0	0
ATOM# 22H	0	0	0.0001
ATOM# 23H	0	0	0
ATOM# 24H	0	0	0
ATOM# 25H	0.0001	0.0001	0.0001
ATOM# 26H	0	0	0
ATOM# 27H	0	0	0
ATOM# 28H	0	0	0
ATOM# 29H	0.0021	0.0021	0.0021

Table S17. Condensed electrophilic Fukui functions (f^{+}) for acephate in gas phase, PCM water, and supermolecule in PCM.

Acephate	Gas Phase	PCM water	Supermolecule in PCM
ATOM# 1S	0.1464	0.1614	0.1810
ATOM# 2P	0.1262	0.1305	0.2042
ATOM# 3O	0.0017	0.0027	0.0010
ATOM# 4O	0.0128	0.0128	0.0316
ATOM# 5O	0.1886	0.1848	0.1559
ATOM# 6N	0.0314	0.0255	0.0226
ATOM# 7C	0.3611	0.3418	0.3080
ATOM# 8C	0.0122	0.0112	0.0077
ATOM# 9C	0.0381	0.0526	0.0106
ATOM# 10C	0.0004	0.0014	0.0060
ATOM# 11H	0.0010	0.0016	0.0001
ATOM# 12H	0.0323	0.0295	0.0087
ATOM# 13H	0.0011	0.0007	0.0018
ATOM# 14H	0.0208	0.0215	0.0311
ATOM# 15H	0.0055	0.0071	0.0049
ATOM# 16H	0.0036	0.0042	0.0011
ATOM# 17H	0.0061	0.0071	0.0154
ATOM# 18H	0.0015	0.0005	0.0004
ATOM# 19H	0.0054	0	0
ATOM# 20H	0.0039	0.0030	0.0002

Table S18. Condensed electrophilic Fukui functions (f^{+}) for methamidophos in gas phase, PCM water, and supermolecule in PCM.

Methamidophos	Gas Phase	PCM water	Supermolecule in PCM
ATOM# 1S	0.5155	0.5222	0.4784
ATOM# 2P	0.2709	0.2541	0.2995
ATOM# 3O	0.0139	0.0133	0.0165
ATOM# 4O	0.0425	0.0421	0.0383
ATOM# 5N	0.0154	0.0134	0.0120
ATOM# 6C	0.0588	0.0600	0.0371
ATOM# 7C	0.0102	0.0120	0.0151
ATOM# 8H	0.0001	0.0003	0.0001
ATOM# 9H	0.0017	0.0008	0.0055
ATOM# 10H	0.0185	0.0218	0.0096
ATOM# 11H	0.0216	0.0309	0.0268
ATOM# 12H	0.0226	0.0216	0.0150
ATOM# 13H	0.0003	0.0002	0.0001
ATOM# 14H	0.0061	0.0046	0.0027

ATOM# 15H	0.0019	0.0026	0.0027
